# The necessity of validity diagnostics when drawing causal inferences from observational data: lessons from a multi-database evaluation of the risk of non-infectious uveitis among patients exposed to Remicade^®^

**DOI:** 10.1186/s12874-024-02428-7

**Published:** 2024-12-27

**Authors:** James Weaver, Erica A. Voss, Guy Cafri, Kathleen Beyrau, Michelle Nashleanas, Robert Suruki

**Affiliations:** 1 Janssen Research & Development LLC, Global Epidemiology Organization, Raritan, NJ USA; 2 Johnson & Johnson MedTech Epidemiology and Real-World Data Sciences, New Brunswick, NJ USA; 3 Johnson & Johnson Global Medical Safety, New Brunswick, NJ USA

**Keywords:** Observational study, Causal inference, Autoimmune disorders, Biologic agents, Non-infectious uveitis

## Abstract

**Background:**

Autoimmune disorders have primary manifestations such as joint pain and bowel inflammation but can also have secondary manifestations such as non-infectious uveitis (NIU). A regulatory health authority raised concerns after receiving spontaneous reports for NIU following exposure to Remicade^®^, a biologic therapy with multiple indications for which alternative therapies are available. In assessment of this clinical question, we applied validity diagnostics to support observational data causal inferences.

**Methods:**

We assessed the risk of NIU among patients exposed to Remicade^®^ compared to alternative biologics. Five databases, four study populations, and four analysis methodologies were used to estimate 80 potential treatment effects, with 20 pre-specified as primary. The study populations included inflammatory bowel conditions Crohn’s disease or ulcerative colitis (IBD), ankylosing spondylitis (AS), psoriatic conditions plaque psoriasis or psoriatic arthritis (PsO/PsA), and rheumatoid arthritis (RA). We conducted four analysis strategies intended to address limitations of causal estimation using observational data and applied four diagnostics with pre-specified quantitative rules to evaluate threats to validity from observed and unobserved confounding. We also qualitatively assessed post-propensity score matching representativeness, and bias susceptibility from outcome misclassification. We fit Cox proportional-hazards models, conditioned on propensity score-matched sets, to estimate the on-treatment risk of NIU among Remicade^®^ initiators versus alternatives. Estimates from analyses that passed four validity tests were assessed.

**Results:**

Of the 80 total analyses and the 20 analyses pre-specified as primary, 24% and 20% passed diagnostics, respectively. Among patients with IBD, we observed no evidence of increased risk for NIU relative to other similarly indicated biologics (pooled hazard ratio [HR] 0.75, 95% confidence interval [CI] 0.38–1.40). For patients with RA, we observed no increased risk relative to similarly indicated biologics, although results were imprecise (HR: 1.23, 95% CI 0.14–10.47).

**Conclusions:**

We applied validity diagnostics on a heterogenous, observational setting to answer a specific research question. The results indicated that safety effect estimates from many analyses would be inappropriate to interpret as causal, given the data available and methods employed. Validity diagnostics should always be used to determine if the design and analysis are of sufficient quality to support causal inferences. The clinical implications of our findings on IBD suggests that, if an increased risk exists, it is unlikely to be greater than 40% given the 1.40 upper bound of the pooled HR confidence interval.

**Supplementary Information:**

The online version contains supplementary material available at 10.1186/s12874-024-02428-7.

## Introduction

Randomized controlled trials (RCT) are the gold standard for estimating causal effects between drug exposures and health outcomes [1–3]. In an ideal, perfectly specified and conducted RCT, the treatment effect is an unbiased estimate of the effect of treatment on the outcome. Randomization ensures balance on baseline observed and unobserved factors that if unbalanced could confound effect estimates [4]. Additionally, RCTs generally evaluate a well-defined study population whose subjects must meet strict inclusion/exclusion criteria applied by trained investigators at enrollment, which selects the subject sample to represent the target population of interest. Further, study outcome case adjudication by trained clinicians reduces or eliminates bias from outcome misclassification.

RCTs are resource intensive and slow in response to urgently needed evidence and medical innovation [3], although the RECOVERY trial demonstrated that rapid RCT execution is possible [5, 6]. They demonstrate strong internal validity through rigorous design and strict inclusion/exclusion criteria, but the tradeoff is reduced external validity and generalizability [7]. Moreover, RCTs are often under powered or of insufficient follow-up time to detect uncommon or longer-term adverse events [8]. Lastly, ethical considerations exclude vulnerable populations from participation [9–11], leaving evidence gaps for patients in need of informed treatment decisions.

So, out of necessity is an opportunity for researchers to attempt to make valid causal inferences from observational data. Following the US 21st Century Cures Act of 2016 [12], real-world evidence (RWE) derived from the analysis of real-world data (RWD) has been increasingly called upon by regulatory authorities for evidentiary needs to complement what is known from clinical trials [13–16].

Despite the promise of using RWE to inform clinical, policy, and regulatory decisions when RCT evidence is unavailable, causal inferences made from RWD have historically been inconsistent or contradictory. For example, in evaluating whether bisphosphonates cause an increased risk of esophageal cancer among patients in the Clinical Practice Research Datalink database, two investigator teams reported and published conflicting results. A comparative cohort study reported no increased risk [17] whereas a nested case-control study reported a 30% increased risk [18]. Such inconsistencies compromise the reliability of and confidence in causal inferences made from RWD^1^.

Nonetheless, recent advances have been promising. Contrary to current guidelines, observational cohort studies found chlorthalidone use was not associated with cardiovascular benefit compared to hydrochlorothiazide [19, 20] and these accurately predicted a subsequently reported RCT assessing the same question [21]. Further, the RCT-DUPLICATE initiative demonstrated high concordance between results from a selected sample of RCTs and corresponding observational study results intended for replication [22]. These advances support continuing health authority commitment to ongoing RWD and RWE policy development [23].

Despite these advances, threats to the validity of causal estimation using observational data persist. Inherent to all observational studies intended to draw causal inferences are risks of bias from observed and unobserved confounding, measurement error such as outcome misclassification, and poor sample representativeness of the target population. In this study, we illustrate the use of diagnostics to assess whether valid causal inferences can be made to address a health authority query on a specific safety concern for a biologic therapy with multiple indications for which many alternative therapies are available. We addressed this concern by conducting an observational, comparative cohort safety study intended to estimate the causal effect on Remicade^®^ on non-infectious uveitis (NIU). To address these concerns in this work, we identified, evaluated, and reduced threats to the validity of our study causal inferences using a set of diagnostics.

### Objectives

We investigated whether exposure to Remicade^®^ caused an increased risk of NIU compared to other biologics within indication-specific study populations across five observational databases. Our analysis underwent four validity diagnostics, supplemented by two qualitative diagnostics. First, we present the clinical study example. Secondly, we detail the validity diagnostics employed to evaluate the analysis’s potential for supporting causal inference. Thirdly, these diagnostics were applied to our observational clinical study. This study showcases how validity diagnostics enhance the credibility of evidence for comparative effect estimation derived from observational data.

## Methods

We designed and conducted an active comparator, new user, PS-matched cohort study [24, 25] to estimate the risk of NIU among new users of Remicade^®^. The pre-specified protocol and complete source code for this study are available at https://github.com/ohdsi-studies/UveitisSafetyEstimation/tree/master/Documents and https://github.com/ohdsi-studies/UveitisSafetyEstimation/. Our observational study adhered to principles of target trial emulation [26, 27] and standardized, comprehensive analyses intended to reduce observational study biases [28]. This was a multi-database study that allows for analysis of diverse patient populations, rare exposures and outcomes, and supports replicability and generalizability [29]. Further, evidence from multi-database studies is strengthened by assessing results consistency across databases.

### Data sources

We conducted the study in five databases, three administrative claims and two electronic health record (EHR) databases, all from the United States (US). The claims databases included Merative™ MarketScan^®^ Commercial Database (CCAE), Optum^®^ de-Identified Clinformatics^®^ Data Mart Database (Clinformatics^®^), IQVIA Pharmetrics Plus (Pharmetrics). The EHR databases included Optum^®^ de-identified Electronic Health Record Dataset (Optum^®^ EHR) and IQVIA Ambulatory EMR (Amb EMR). These five US databases provide multiple perspectives on the study populations given variation in population composition and data capture process by database. Detailed database descriptions are available in Appendix 1.

The databases were standardized in structure and content into the Observational Medical Outcomes Partnership (OMOP) Common Data Model (CDM) [30, 31] which is maintained by the Observational Health Data Sciences and Informatics (OHDSI) community. This standardization allows the strictly consistent application of analytic routines across multiple, disparate databases that eliminates variability of cohort definitions, variable definitions, and analytic implementation.

### Study populations

We assessed four non-mutually exclusive populations that are indicated for Remicade^®^: patients with inflammatory bowel conditions Crohn’s disease or ulcerative colitis (IBD), ankylosing spondylitis (AS), psoriatic conditions plaque psoriasis or psoriatic arthritis (PsO/PsA), and rheumatoid arthritis (RA). The indication cohort definitions are fully specified in Appendix 2. Comprehensive clinical characterization of the Remicade^®^-indicated study populations is available for review at an interactive web application at https://results.ohdsi.org/app/15_UveitisSafetyIndications.

#### Exposures

Within each study population we compared new users of a target exposure to new users of comparator exposures, that we refer to as the target and comparator cohorts. In the IBD, AS, PsO/PsA study populations, the target cohorts consisted of patients newly exposed to Remicade^®^. In the RA study population, the target cohort consisted of patients newly exposed to Remicade^®^ concurrently exposed to methotrexate^2^ [32]. We compared the Remicade^®^ target cohorts to the comparator cohorts, which differed by study population. We defined the comparator cohorts by new use of one of several alternative therapies indicated for IBD, AS, PsO/PsA, or RA and are listed in Table 1. The comparator exposures are biologics approved by the US Food and Drug Administration (FDA) for treatment of the indication study populations. Further, we excluded specific exposures for which there exists evidence of an increased or decreased risk for NIU. Specifically, we excluded etanercept and adalimumab from all comparator cohorts. Etanercept is known to increase the risk of uveitis and adalimumab is approved as a treatment for uveitis, as well as known to decrease the risk of uveitis [33–35].

**Table 1:** Exposure comparisons by indication

Study population	Target cohorts	Target approval date	Comparator Cohorts	Comparator with earliest approval date	Comparator with latest approval date
Irritable bowel diseases (Crohn’s disease or ulcerative colitis)	Remicade^®^	8/24/1998	-golimumab -certolizumab pegol -ustekinumab -vedolizumab	4/22/2008 (certolizumab pegol)	9/26/2016 (ustekinumab)
Ankylosing Spondylitis	Remicade^®^	12/17/2004	-golimumab -certolizumab pegol -ixekizumab -secukinumab	4/24/2009 (golimumab)	8/26/2019 (ixekizumab)
Plaque psoriasis or psoriatic arthritis	Remicade^®^	5/13/2005	-golimumab -certolizumab pegol -guselkumab -risankizumab -tildrakizumab -brodalumab -ixekizumab -secukinumab -ustekinumab	4/24/2009 (golimumab)	4/23/2019 (risankizumab)
Rheumatoid arthritis	Remicade^®^ with concurrent methotrexate*	11/10/1999	-certolizumab pegol -tocilizumab	5/13/2009 (certolizumab pegol)	1/11/2010 (tocilizumab)

* Remicade^®^ should be administered in combination with methotrexate for the treatment of rheumatoid arthritis

Table 1 presents the comparator drugs used as the reference to which the target cohorts were compared for each indication. The target cohort population was limited to index exposures after the earliest date of approval by the FDA for the drugs included in the comparator cohort. Patients aged at least 18 years at the time of index and with at least 365 days of prior observation were eligible to participate in both the target and comparator cohorts. Additionally, target cohort patients were required to be naïve to biologics and infliximab biosimilars. All patients in the target and comparator cohorts were required to have no previous exposure to the medications listed as restrictions in Table 2. The detailed target and comparator cohort definitions are in Appendix 2.

**Table 2:** Exposure cohort restrictions and right-censoring criteria

Indication	Cohort type	Exposures	Restrictions	Right-censoring criteria*
Irritable bowel diseases (Crohn’s disease or ulcerative colitis)	Target	Remicade^®^	-TNFαi^1^ except infliximab -infliximab biosimilars -interleukin inhibitors^2^ -vedolizumab -natalizumab	-adalimumab -certolizumab pegol -etanercept -golimumab -vedolizumab -natalizumab -interleukin inhibitors^2^
Irritable bowel diseases (Crohn’s disease or ulcerative colitis)	Comparator	golimumab, certolizumab pegol, ustekinumab, or vedolizumab	-TNFαi^1^ except certolizumab pegol and golimumab -natalizumab	-infliximab -adalimumab -etanercept -natalizumab -interleukin inhibitors^2^ except ustekinumab
Ankylosing spondylitis	Target	Remicade^®^	-TNFαi^1^ except infliximab -infliximab biosimilars -interleukin inhibitors^2^	-adalimumab -etanercept -golimumab -certolizumab pegol -interleukin inhibitors^2^
Ankylosing spondylitis	Comparator	golimumab, certolizumab pegol, ixekizumab, or secukinumab	-TNFαi^1^ except certolizumab pegol and golimumab -interleukin inhibitors^2^	-infliximab -adalimumab -etanercept -interleukin inhibitors^2^ except ixekizumab and secukinumab
Plaque psoriasis or psoriatic arthritis	Target	Remicade^®^	-TNFαi^1^ except infliximab -infliximab biosimilars -interleukin inhibitors^2^	-adalimumab -etanercept -certolizumab pegol -golimumab -interleukin inhibitors^2^
Plaque psoriasis or psoriatic arthritis	Comparator	golimumab, certolizumab pegol, guselkumab, risankizumab, tildrakizumab, brodalumab, ixekizumab, secukinumab, or ustekinumab	-infliximab -adalimumab -certolizumab pegol -etanercept -golimumab -Interleukin inhibitors^2^	-infliximab -adalimumab -etanercept -interleukin inhibitors^2^ except brodalumab, guselkumab, ixekizumab, Risankizumab, secukinumab, tildrakizumab and ustekinumab
Rheumatoid Arthritis	Target	Remicade^®^ & Methotrexate*	-TNFαi^1^ -interleukin inhibitors^2^ -abatacept	-adalimumab -certolizumab pegol -etanercept -golimumab -abatacept -interleukin inhibitors^2^
Rheumatoid Arthritis	Comparator	certolizumab pegol or tocilizumab	-TNFαi^1^ -interleukin inhibitors^2^ -abatacept	-infliximab -adalimumab -etanercept -golimumab -abatacept -interleukin inhibitors^2^ except tocilizumab

Key: TNFαi: tumor necrosis factor α inhibitors

*Right-censoring criteria for all exposure cohorts includes exposure discontinuation and database discontinuation

1: adalimumab, certolizumab pegol, etanercept, golimumab, infliximab

2: anakinra, basiliximab, brodalumab, canakinumab, daclizumab, guselkumab, ixekizumab, rilonacept, risankizumab, sarilumab, sarilumab, secukinumab, siltuximab, tildrakizumab, tocilizumab, ustekinumab

Remicade^®^ dosage varies by indication^3^. Because our study is stratified by indication, it is unlikely Remicade® dosage variation will violate the consistency assumption for causality (i.e., each patient receives the same version of treatment, or if multiple versions of a treatment do exist, then they have the same effect on the outcome) [36].

#### Outcomes

Phenotyping is the process by which the physiological, clinical description of a medical condition is translated into a computable algorithm designed to identify patients with the condition from an observational data source [37, 38]. We applied a novel phenotyping [39] and outcome validation [40, 41] approach to developing and evaluating a phenotype algorithm for patients with NIU with intent to minimize misclassification. In studies that use ratio effect estimates such as ours, low outcome sensitivity is tolerable provided specificity is high to obtain an unbiased estimate of treatment effect [42].

The novel outcome validation method we used builds a probabilistic reference standard rather than using deterministic medical chart adjudication. We fit a diagnostic predictive model that assigns case probabilities to a large reference set against which we compared patients returned by our candidate outcome phenotype algorithms. Case probabilities are assigned to cases and non-cases which allowed us to populate a full confusion matrix with the sums of conditional probabilities to compute all misclassification metrics. Briefly, NIU is intraocular inflammation, characterized by inflammation of the uvea in the absence of infection. We developed and evaluated three outcome definitions:

- Broad – first occurrence of a NIU code.
- Narrow – first occurrence of a NIU code with a second NIU code occurrence between 31 days and 365 days relative to first occurrence.
- Primary – [first occurrence of a NIU code with a second NIU code occurrence between 31 days and 365 days relative to first occurrence] OR [first occurrence of a NIU code during an ophthalmology visit].

We ultimately used the primary definition in our comparative study given its high specificity and its favorable tradeoff between sensitivity and patient count compared to the other definitions. The full clinical description of NIU, the full code list and temporal logic specifications of our three candidate algorithms, and the results of our phenotyping development and evaluation are reported in Appendix 3. We comprehensively characterized our candidate NIU definitions which are available at https://results.ohdsi.org/app/14_UveitisSafetyOutcomes. Misclassification errors for the primary outcome definition is reported in Table 3. It was on the basis of the phenotype evaluation results reported in Appendix 3 that we decided to use the primary NIU definition.

**Table 3:** Confusion matrix contingency cell counts and misclassification errors for the primary non-infectious uveitis outcome definition across databases

Database	TP	TN	FP	FN	Sensitivity	Specificity	PPV	NPV
Amb EMR	496	1,412,594	241	465	0.516129	0.999829	0.672999	0.99967
Pharmetrics	2249	1,967,965	1244	6378	0.260577	0.999368	0.643573	0.996769
Optum^®^ EHR	1369	1,946,703	317	3271	0.295043	0.999838	0.811981	0.998323
Clinformatics^®^	6007	1,658,007	1673	6725	0.471762	0.998992	0.782031	0.99596
CCAE	4044	1,912,446	949	6283	0.391595	0.999504	0.809934	0.996725

Key –Amb EMR: IQVIA Ambulatory Electronic Medical Records, Pharmetrics: IQVIA Adjudicated Health Plan Claims Data, Optum^®^ EHR: Optum^®^ De-Identified Electronic Health Record, Clinformatics^®^: Optum^®^ De-Identified Clinformatics^®^ Data Mart Database, CCAE: Merative™ MarketScan^®^ Commercial Database, TP: true positives, TN: true negatives, FP: false positives, FN: false negatives, Sensitivity = TP/(TP + FN), Specificity = TN/(TN + FP), PPV = positive predictive value = TP/(TP + FP), NPV = negative predictive value = TN/(TN + FN), Primary outcome definition: [first occurrence of a NIU code with a second NIU code occurrence between 31 days and 365 days relative to first occurrence] OR [first occurrence of a NIU code during an ophthalmology visit]

### Cohort study analysis specifications

We fit a large-scale PS model (LSPS) [43, 44] to ensure baseline balance on directly and indirectly measured covariates [45, 46] between the target and comparator cohorts. The PS was calculated for each patient as the predicted probability of target exposure status from an L1 regularized logistic regression model, fit with a Laplace prior where the regularization hyperparameter was selected by optimizing the likelihood in a 10-fold cross validation with a starting variance of 0.01 and a tolerance of 2*10^− 7^ [47]. PS model input covariates included demographics, several risk indices, and code occurrence-based, baseline covariates for all medical diagnoses, drug exposures, procedure occurrences, device use, and laboratory measurements (Appendix 4). Our primary PS adjustment strategy matched target to comparator patients using a 1:10 maximum variable ratio matching approach and used a greedy matching algorithm that applied a caliper of 0.2 of the standard deviation on the logit scale of the PS distribution [48].

We defined the ‘on-treatment’ time-at-risk (TAR) as the day after index until the end of a period of inferred persistent exposure. This allowed no more than a 90-day persistence window between successive exposures plus a 90-day added surveillance to the last exposure date. We chose this persistence window based on recommended administration frequency [49] and an empirical assessment of the durations between subsequent administrations for the drugs in three data sources included in this study. The days distribution between successive exposures showed that 75% of sequential administration records occurred within 90 days for all drugs in all databases except for ustekinumab in Optum EHR^®^. Further, 90% of exposure records occurred within 90 days for most drugs except for ustekinumab, which may have time-at-risk right-censored early for approximately 10% of patients. Appendix 5 reports time distributions between subsequent exposures for the study drugs. This approach was consistent with safety follow-up in registry regulatory safety studies for biologics marketed by the sponsor [49]. Additionally, we right-censored ‘on-treatment’ TAR at an exposure to a comparison drug, adalimumab, or etanercept; for the target cohorts, exposure was censored on other TNF alpha inhibitor (TNFαi) or interleukin inhibitors and the comparator cohorts, exposure was censored at the exposures listed in Table 2.

Within each database and study population, we fit a Cox proportional-hazards (PH) regression model conditioned on PS-matched sets with Remicade^®^ treatment status as the explanatory variable to model the time to the first ever NIU occurrence relative to the comparator group. This requirement excluded patients with a pre-index NIU event from the analysis.

In addition to the NIU outcome of interest, we also executed each study comparison against a set of 86 negative control outcomes to identify and correct for unobserved confounding and design or analytic misspecification [50]. Negative control outcomes are conditions known not to be causally associated with the target or comparator cohort exposures. Negative controls were selected by a semi-automated process that identifies conditions with no evidence of causal drug effects per spontaneous reports, published literature, and product labels [51]. Because of the a priori assertion of no target or comparator effect on the negative control outcomes, we assume the difference between hypothetical null (hazard ratio [HR] = 1) and the observed effect on a negative control is considered residual systematic error from unmeasured sources. The set of negative controls outcomes are in Appendix 6. We calibrated the NIU hazard ratios against the empirical null distribution to adjust for observed residual bias and reported calibrated hazard ratios (cHR) as the effect estimate.

In addition to the primary analysis described above, we included secondary analyses with 1:1 PS matching and an ‘intention-to-treat’ (ITT) TAR. The ITT TAR began on the day after index and ended at the end of observation time in the database and was not right censored at discontinuation or exposure to other drugs.

Our two PS matching strategies (1:10, 1:1), two TAR risk definitions (‘on-treatment’, ‘ITT’), four comparisons (Remicade^®^ vs. comparator in IBD, AS, PsO/PsA, and RA study populations), by five databases (CCAE, Clinformatics^®^, Pharmetrics, Optum^®^ EHR, and Amb EMR) resulted in 80 individual analyses, each intended to produce a single effect estimate. Twenty of these analyses were designated as primary (1:10 matching strategy, ‘on-treatment’ TAR, by four comparisons, and by five databases).

For each study population comparative analysis that passed diagnostics, we calculated the heterogeneity of hazard ratios across databases using the I^2^ metric and performed a meta-analysis using a DerSimonian-Laird estimate of the random effects variance [52]. We computed meta-analytic effect estimates when estimates of heterogeneity across databases were sufficiently low (I^2^ < 40%). Meta-analytic results from our primary analysis were our main source of statistical inference from which we drew causal inference conclusions. Where meta-analytic estimates were unavailable because of failing diagnostics or unacceptable heterogeneity, we reported and interpreted database-specific estimates.

### Evidence validity diagnostics

The target estimand in this study is the average treatment effect in the overlap (ATO) population [53]. One key assumption for causal inference from a potential outcomes framework is exchangeability [54]. In the context of estimating the average treatment effect among the treated (ATT) (and the ATO using PS matching with a caliper) we assume partial exchangeability, the potential outcome under no treatment must be unrelated to treatment assignment conditional on measured covariates [36, 53]. The PS is a balancing score, such that the distribution of observed baseline covariates will be equivalent between target and comparator patients with similar PS values, and if strong ignorability with partial exchangeability holds then treatment assignment is unrelated to the potential outcome under no treatment conditional on the PS [43]. PS matching is used to approximate exchangeability between exposed patient cohorts that have been selectively assigned treatments during routine clinical care. Exchangeable target and comparator cohorts are those where exposure status is the only difference between them, where we can attribute any difference in outcome occurrence to exposure status only [55].

For each analysis in each database intended to generate an effect estimate, we applied the following validity diagnostics to determine whether we could report the result as reliable.

#### Empirical equipoise

Empirical equipoise is a diagnostic related to partial exchangeability. Specifically, target and comparator cohorts with similar PS distributions, or a high degree of overlap, will have similar baseline covariate distributions on average. Further, these patient cohorts will resemble each other on observed baseline covariates including confounders, thereby making it more likely that the partial exchangeability assumption has been met. After fitting the PS model, plotting the PS distribution stratified by exposure status can help assess partial exchangeability. By calculating the proportion of study population patients with PS overlap near equipoise (PS = 0.5), we can appraise comparability appropriateness before applying any statistical balancing techniques to strengthen exchangeability. A patient is in empirical equipoise if their preference score (a transformation of the PS that normalizes for exposure cohort size imbalances) is between 0.3 and 0.7 of the preference score distribution [56]. If the proportion of patients in empirical equipoise was less than 35% in an analysis, then it failed the equipoise diagnostic. We were more liberal than the 50% threshold proposed by Walker [56] because we prioritized bias reduction and internal validity over initial comparability assessment.

#### Covariate balance

Covariate balance is another diagnostic related to partial exchangeability. Conditional on the PS, patients of different exposure status should have similar distributions of baseline covariates. This assertion requires empirical confirmation to meet the assumption that treatment effect estimates are only valid only if patients in the two exposure cohorts have similar distributions of observed baseline covariates [57]. In the sample of PS-matched patients, we assessed baseline covariate distribution similarity by calculating and plotting the absolute standardized difference (ASD) [57] of every covariate before and after applying PS matching. For binary covariates, the ASD is the absolute prevalence difference of a covariate in units of the pooled standard deviation and is insensitive to sample size. We considered after matching ASDs less than 0.1 to indicate a negligible difference between cohorts in a pairwise comparison [57]. If any covariate in a comparison had an ASD greater than or equal to 0.1, then the analysis failed the covariate balance diagnostic.

#### Expected absolute standardized error

The expected absolute standardized error (EASE) metric detects and quantifies residual bias from unobserved sources, which relates to the assumption of partial exchangeability. To compute EASE, we first generate a residual systematic error distribution using effect size estimates for negative controls, assuming this distribution follows a normal distribution. We fit this distribution similarly to the random-effects component in a meta-analysis, capturing deviations from the null that are not attributed to random error (as indicated by estimated systematic errors) [58, 59]. EASE then summarizes this systematic error distribution by integrating over its absolute values. An EASE of 0 suggests that the variance in negative control estimates is fully explained by random error, indicating the absence of systematic error. We considered analyses where EASE was greater than 0.25 to have failed the diagnostic. When EASE = 0.25 and systematic error is centered on 0, a true relative risk of 1 has a 95% probability of being observed between 0.54 and 1.85 due to systematic error. Although empirical calibration could statistically restore nominal operating characteristics, we decided EASE > 0.25 identified unacceptable design operating characteristics even after PS adjustment.

#### Non-zero event counts

For a HR to be estimated from a Cox PH model, outcome occurrences during the TAR for both target and comparator cohorts in the analysis had to be greater than zero. Otherwise, the HR would approach negative or positive infinity, which is not a valid estimate of a causal effect. Analyses where target and/or comparator TAR outcome occurrence counts were zero failed this diagnostic.

### Representativeness

In establishing valid causal estimates from observational data, patient restriction from the original study population is sometimes required, for example, when patients are excluded after PS matching or for having an outcome occur before index. This practice is often necessary to ensure the interval validity of the study, but it can be at odds with representativeness. While it has been argued that representativeness may not be essential for scientific study [60], the extent to which the characteristics distribution of the restricted analytic cohort differ from that of the original study population can be assessed empirically. We assessed the extent to which baseline characteristics of the after-matching target cohort are like those of the initial target cohort. We evaluated covariate similarity between the two cohorts by plotting the prevalence of all baseline covariates and calculating ASDs [61]. Note that representativeness was assessed qualitatively with no set threshold for meeting a representativeness criterion.

We set the thresholds for the empirical equipoise, covariate balance, and expected absolute standardized error somewhat arbitrarily, but we assert that a critical feature of validity diagnostics is to set thresholds a priori and adhere to them strictly to avoid investigator bias from post-hoc analyses conditional on preliminary results. Our pre-specified protocol was posted at: https://github.com/ohdsi-studies/UveitisSafetyEstimation/tree/master/Documents.

## Results

Our full diagnostics and clinical results are publicly available for review in the **Estimation Diagnostics Explorer** interactive web application available at https://data.ohdsi.org/UveitisSafetyEstimation/. In the ‘Explore results’ tab on the left panel, a user can select a target cohort, comparator cohort, data source, and analysis variant to display a results table that includes database-specific and meta-analytic results for the selection. Results in the table that did not pass all validity diagnostics are blinded as ‘NA’ to discourage the investigators and reviewers from interpreting flawed causal estimates. By clicking a row in the table, a set of diagnostics results associated with the row estimate is presented including exposure and event counts, minimum detectable relative risk, attrition tables, representativeness statistics, PS diagnostics, covariate balance, and the empirical null distribution.

### Evidence evaluation

We subjected 80 analyses to validity diagnostics of which 19 (24%) passed. Of the 20 analyses designated as primary, 4 (20%) passed diagnostics. Of the 34 analyses that passed three diagnostics, most failed to achieve covariate balance where all covariates had an ASD < 0.1. Of the 15 analyses that passed two diagnostics, all failed covariate balance and most failed equipoise.

Table 4 reports the 20 primary analyses with associated diagnostics results. Each row represents a single target versus comparator comparison within an indicated study population per database. Further, each row includes columns indicating values for each validity diagnostic and an indicator for whether the diagnostic passed the pre-specified criteria. For example, the first row represents the comparison between new users of Remicade^®^ and new users of certolizumab pegol, golimumab, ixekizumab, or secukinumab among patients with AS in the Amb EMR database. In this analysis, we observed no NIU events during either exposure cohort TAR (failed diagnostic), the maximum ASD was 0.302 (failed diagnostic), the proportion of patients in empirical equipoise was 0.531 (passed diagnostic), and the EASE metric was 0.976 (failed diagnostic). Consequently, this analysis passed 1 of 4 diagnostics therefore the effect estimate was not reviewed because it could not be interpreted as causally valid.

**Table 4:** Diagnostic results for primary analyses, rows bolded passed four validity diagnostics

Study pop.	Database	Target	Comparator	T events	C events	0 event Pass	Max ASD	ASD Pass	Equipoise	Equipoise Pass	EASE	EASE Pass	Total Passed
AS	Amb EMR	Remicade^®^	AS comparator	0	0	0	0.302	0	0.531	1	0.976	0	1
AS	Pharmetrics	Remicade^®^	AS comparator	6	9	1	0.157	0	0.405	1	0.044	1	3
AS	Optum^®^ EHR	Remicade^®^	AS comparator	< 5	6	1	0.218	0	0.638	1	0.233	1	3
AS	Clinformatics^®^	Remicade^®^	AS comparator	< 5	< 5	1	0.272	0	0.417	1	0.172	1	3
AS	CCAE	Remicade^®^	AS comparator	< 5	6	1	0.239	0	0.434	1	0.180	1	3
IBD	Amb EMR	Remicade^®^	IBD comparator	< 5	< 5	1	0.09	1	0.422	1	0.284	0	3
**IBD**	**Pharmetrics**	**Remicade** ^**®**^	**IBD comparator**	**12**	**42**	**1**	**0.047**	**1**	**0.431**	**1**	**0.074**	**1**	**4**
**IBD**	**Optum** ^**®**^ ** EHR**	**Remicade** ^**®**^	**IBD comparator**	**< 5**	**7**	**1**	**0.055**	**1**	**0.480**	**1**	**0.087**	**1**	**4**
IBD	Clinformatics^®^	Remicade^®^	IBD comparator	6	15	1	0.105	0	0.412	1	0.040	1	3
**IBD**	**CCAE**	**Remicade** ^**®**^	**IBD comparator**	**10**	**18**	**1**	**0.071**	**1**	**0.387**	**1**	**0.107**	**1**	**4**
PsO/PsA	Amb EMR	Remicade^®^	PsO/PsA comparator	0	0	0	0.145	0	0.254	0	0.344	0	0
PsO/PsA	Pharmetrics	Remicade^®^	PsO/PsA comparator	< 5	10	1	0.132	0	0.155	0	0.178	1	2
PsO/PsA	Optum^®^ EHR	Remicade^®^	PsO/PsA comparator	6	< 5	1	0.099	1	0.306	0	0.246	1	3
PsO/PsA	Clinformatics^®^	Remicade^®^	PsO/PsA comparator	0	7	0	0.199	0	0.171	0	0.110	1	1
PsO/PsA	CCAE	Remicade^®^	PsO/PsA comparator	< 5	9	1	0.167	0	0.147	0	0.010	1	2
RA	Amb EMR	Remicade^®^(m)	RA comparator	< 5	0	0	0.127	0	0.445	1	0.307	0	1
RA	Pharmetrics	Remicade^®^(m)	RA comparator	< 5	6	1	0.179	0	0.352	1	0.158	1	3
**RA**	**Optum** ^**®**^ ** EHR**	**Remicade** ^**®**^ **(m)**	**RA comparator**	**5**	**< 5**	**1**	**0.097**	**1**	**0.558**	**1**	**0.141**	**1**	**4**
RA	Clinformatics^®^	Remicade^®^(m)	RA comparator	< 5	8	1	0.252	0	0.363	1	0.034	1	3
RA	CCAE	Remicade^®^(m)	RA comparator	< 5	8	1	0.151	0	0.508	1	0.070	1	3

Key: <5 = a censored value between 1 and 4; Amb EMR = IQVIA Ambulatory Electronic Medical Records; AS comparator = certolizumab pegol, golimumab, ixekizumab, or secukinumab; AS = ankylosing spondylitis; ASD = absolute standardized difference; CCAE = Merative™ MarketScan^®^ Commercial Database; Clinformatics^®^ = Optum^®^ De-Identified Clinformatics^®^ Data Mart Database; EASE = expected absolute systematic error; IBD comparator = golimumab, certolizumab pegol, ustekinumab, or vedolizumab; IBD = irritable bowel diseases (Crohn’s disease or ulcerative colitis); Optum^®^ EHR = Optum^®^ De-Identified Electronic Health Record; Pharmetrics = IQVIA Adjudicated Health Plan Claims Data; PsO/PsA comparator = golimumab, certolizumab pegol, guselkumab, risankizumab, tildrakizumab, brodalumab, ixekizumab, secukinumab, or ustekinumab; PsO/PsA = psoriatic conditions plaque psoriasis or psoriatic arthritis; RA comparator = certolizumab pegol or tocilizumab; RA = rheumatoid arthritis; Remicade^®^(m) = Remicade^®^ exposure with concurrent methotrexate; Study pop. = study population

Rows that are bolded indicate analyses that passed the four validity diagnostics. In the IBD study population, primary analyses in Pharmetrics, Optum^®^ EHR, and CCAE passed the four diagnostics meaning we reviewed the estimates and could interpret them as causally valid. In the RA study population, the primary analysis in Optum EHR^®^ passed the four diagnostics meaning we could interpret the estimate as causally valid.

**Fig. 1:**
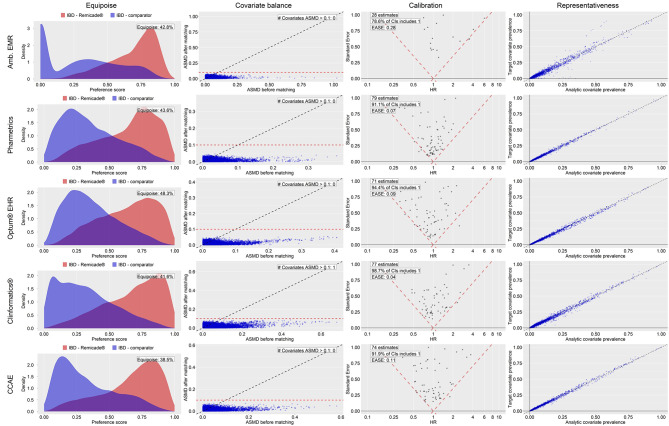
Empirical equipoise, covariate balance, empirical calibration validity diagnostics and representativeness for IBD primary analysis Key: Amb EMR = IQVIA Ambulatory Electronic Medical Records; ASMD = absolute standardized mean difference; CCAE = Merative™ MarketScan^®^ Commercial Database; CI = Confidence Interval; Clinformatics^®^ = Optum^®^ De-Identified Clinformatics^®^ Data Mart Database; EASE = expected absolute systematic error; HR = Hazard ratio; IBD = irritable bowel diseases (Crohn’s disease or ulcerative colitis); IBD comparator = golimumab, certolizumab pegol, ustekinumab, or vedolizumab; Optum^®^ EHR = Optum^®^ De-Identified Electronic Health Record; Pharmetrics = IQVIA Adjudicated Health Plan Claims Data; Remicade^®^(m) = Remicade^®^ exposure; Target covariate prevalence = prevalence of baseline covariates in the initial Remicade^®^ exposure cohort before study design restrictions were applied; Analytic covariate prevalence = prevalence of baseline covariates in Remicade^®^ exposure cohort after study design restrictions were applied (i.e., PS matching)

Figure 1 depicts the preference score overlap, covariate balance plot, the empirical null distribution, and representativeness diagnostics (columns) for the five databases (rows) for the comparisons in the IBD study population. Corresponding to the bolded IBD rows in Table 4, the Fig. 1 rows for Pharmetrics, Optum^®^ EHR, and CCAE present plots that passed each diagnostic. In the Pharmetrics row, empirical equipoise is 43.6% (greater than the 35% pre-specified threshold), 0 covariates had an ASD > 0.1, and EASE was 0.07 (less than the 0.25 pre-specified threshold). Lastly, the representativeness plot displays high concordance between covariate prevalence of the initial target Remicade^®^ cohort (*n* = 22,451) [see Fig. 3] and the after matching Remicade^®^ cohort (*n* = 10,169) from which we estimated the effect on NIU. The close distribution of data points about the diagonal indicates that the prevalence of baseline covariates between the target and after matching cohort were similar, suggesting that patient attrition from PS matching did not substantially alter the composition of the target population. By contrast, the representativeness plot for Amb EMR suggests greater differences between the target and after matching cohort in this database. We observed similar passed diagnostic results in the Fig. 1 rows for the Optum^®^ EHR and CCAE databases. It is worth noting that Clinformatics^®^ failed diagnostics based on ASD = 0.1 for one covariate (an observation of ‘Requires Bacillus Calmette-Guerin vaccination’ in 365 days before and including index). This covariate was of low prevalence before and after matching, so despite difference across cohorts, may not have a strong confounding impact on the effect estimate were it also associated with the outcome. But strict adherence to a priori defined thresholds dictate a diagnostics failure.

**Fig. 2:**
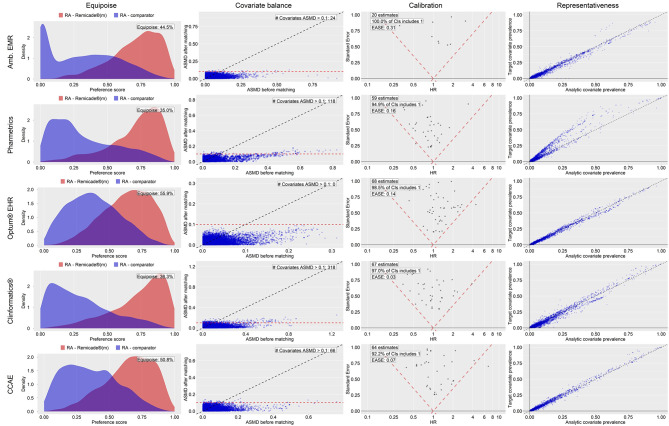
Empirical equipoise, covariate balance, empirical calibration validity diagnostics and representativeness for RA primary analysis Key – Target: patients with inflammatory bowel diseases newly exposed to Remicade^®^, Comparator: patients with inflammatory bowel diseases newly exposed to [golimumab, certolizumab pegol, ustekinumab, or vedolizumab], CCAE: Merative™ MarketScan^®^ Commercial Database, Optum^®^ EHR = Optum^®^ De-Identified Electronic Health Record; Pharmetrics = IQVIA Adjudicated Health Plan Claims Data

Figure 2 depicts the same diagnostics information as Fig. 1 but for the comparisons in the RA study population. Corresponding to the bolded RA row in Table 4, the Fig. 2 row for Optum^®^ EHR presents plots that passed each diagnostic. Empirical equipoise is 55.9% (greater than the 35% pre-specified threshold), 0 covariates had an SMD > 0.1, and EASE was 0.14 (less than the 0.25 pre-specified threshold). Lastly, representativeness was considered acceptable by the high concordance between covariate prevalence of the initial target Remicade^®^ with methotrexate cohort (*n* = 4,173) and the analytic Remicade^®^ with methotrexate cohort (*n* = 2,700) from which we estimated the effect on NIU. Of note is that in Pharmetrics, the prevalence of baseline characteristics was greater in the Remicade^®^ with concurrent methotrexate initial target cohort than after PS matching, suggesting that attrition from PS matching may have selectively excluded patients of greater comorbidity.

**Fig. 3:**
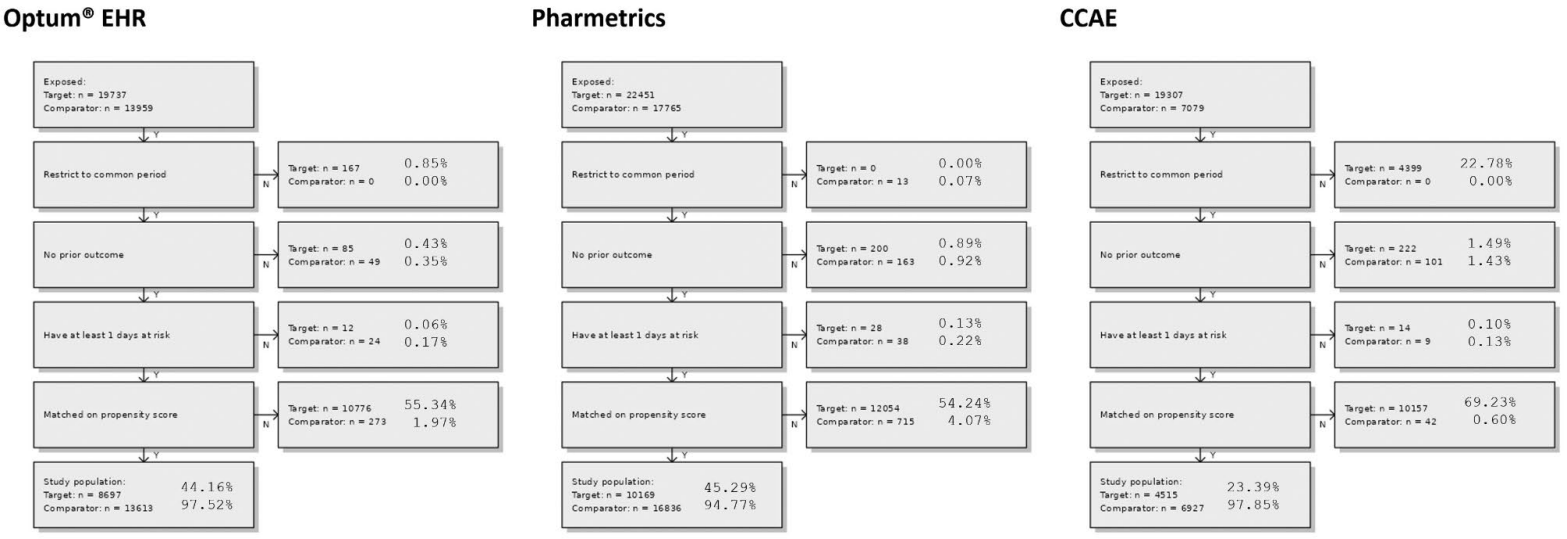
Attrition diagrams for inflammatory bowel diseases (IBD); patient attrition counts and proportions after sequential design choices applied Key: Amb EMR = IQVIA Ambulatory Electronic Medical Records; ASMD = absolute standardized mean difference; CCAE = Merative™ MarketScan^®^ Commercial Database; CI = Confidence Interval; Clinformatics^®^ = Optum^®^ De-Identified Clinformatics^®^ Data Mart Database; EASE = expected absolute systematic error; HR = Hazard ratio; Optum^®^ EHR = Optum^®^ De-Identified Electronic Health Record; Pharmetrics = IQVIA Adjudicated Health Plan Claims Data; RA = rheumatoid arthritis; RA comparator = certolizumab pegol or tocilizumab; Remicade^®^(m) = Remicade^®^ exposure with concurrent methotrexate; Target covariate prevalence = prevalence of baseline covariates in the initial Remicade^®^ exposure cohort before study design restrictions were applied; Analytic covariate prevalence = prevalence of baseline covariates in Remicade^®^ exposure cohort after study design restrictions were applied (i.e., PS matching)

Appendix 7 reports diagnostic results for secondary analyses in all study populations. Twelve secondary analyses passed diagnostics in the IBD study population across multiple data sources, some of which contributed to meta-analytic results. Three secondary analyses passed diagnostics in the RA study population, all in Optum^®^ EHR. The figures supporting diagnostic results from secondary analyses are available in the **Estimation Diagnostics Explorer**.

### Clinical results

#### Primary findings

##### IBD

Figure 2 reports attrition counts for design restrictions of the primary analysis in the databases that passed diagnostics (Optum^®^ EHR, Pharmetrics, CCAE). For example, in Optum EHR^®^ the initial target cohort included 19,737 patients (new users of Remicade^®^ with IBD, definition in Appendix 2). After restricting to calendar time when target and comparator patients were both observed, excluding patients with a prior outcome and who had no time-at-risk, and excluding patients not matched on the PS, the final after matching target cohort included 8,697 patients (44% of initial target population). In Pharmetrics and CCAE the after matching population was 45% and 23% of the initial target population, respectively. Note that the initial target cohort size was greater than the initial comparator cohort size (e.g., 19,307 vs. 7,079 in CCAE, Fig. 2). The high attrition from PS matching results from our applying 1:10 variable ratio target to comparator PS matching, where the initial target cohort patient count is greater than that of the comparator. This could impact representativeness, although our representativeness assessment showed few observable characteristic distribution differences between the initial and after matching target cohorts.

**Fig. 4:**
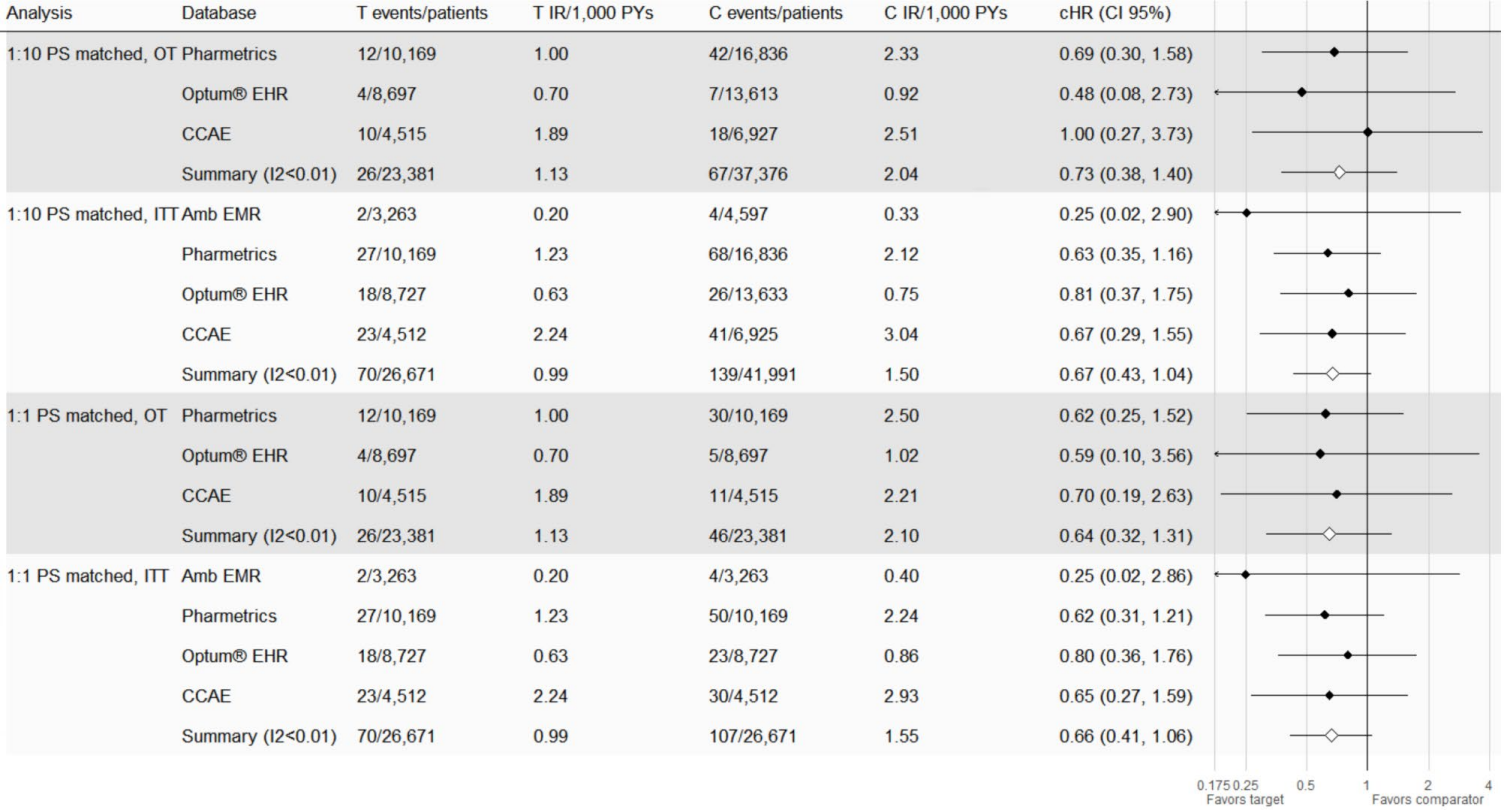
Risk of non-infectious uveitis (NIU) among patients with inflammatory bowel diseases (IBD) Key – PS: propensity score, OT: on-treatment, ITT: intention-to-treat, T: Remicade^®^ new users with IBD, C: golimumab, certolizumab pegol, ustekinumab, or vedolizumab new users with IBD, IR: incidence rate, PYs: person-years, CCAE: Merative™ MarketScan^®^ Commercial Database, Optum^®^ EHR: Optum^®^ De-Identified Electronic Health Record, Pharmetrics: IQVIA Adjudicated Health Plan Claims Data

Figure 4 reports the primary analysis after matching target and comparator patient counts, event counts, incidence rates per 1000 person-years (IR/1k PYs) and calibrated hazard ratios with 95% confidence intervals (cHR [95% CI]) for database that passed diagnostics (Optum^®^ EHR, Pharmetrics, CCAE) and the meta-analytic estimate. Database-specific IRs ranged from 0.70 to 2.51/1k PYs classifying NIU in the exposed populations with IBD as rare to uncommon [62]. The pooled IRs were 1.13 and 2.04/1k PYs (uncommon) for the target and comparator cohorts, respectively. The database-specific estimates ranged from 0.48 (Optum^®^ EHR) to 1.00 (CCAE). The meta-analytic result failed to reject the null hypothesis of no effect and indicated that Remicade^®^ was not associated with an increased risk of NIU compared to [golimumab, certolizumab pegol, ustekinumab, or vedolizumab] during the on-treatment time at risk (cHR 0.73 95% CI 0.38, 1.40). If an increased risk of NIU caused by Remicade^®^ in the IBD study population exists, it is unlikely to be greater than 40%, given the 1.40 upper bound of the pooled cHR confidence interval.

##### RA

**Fig. 5:**
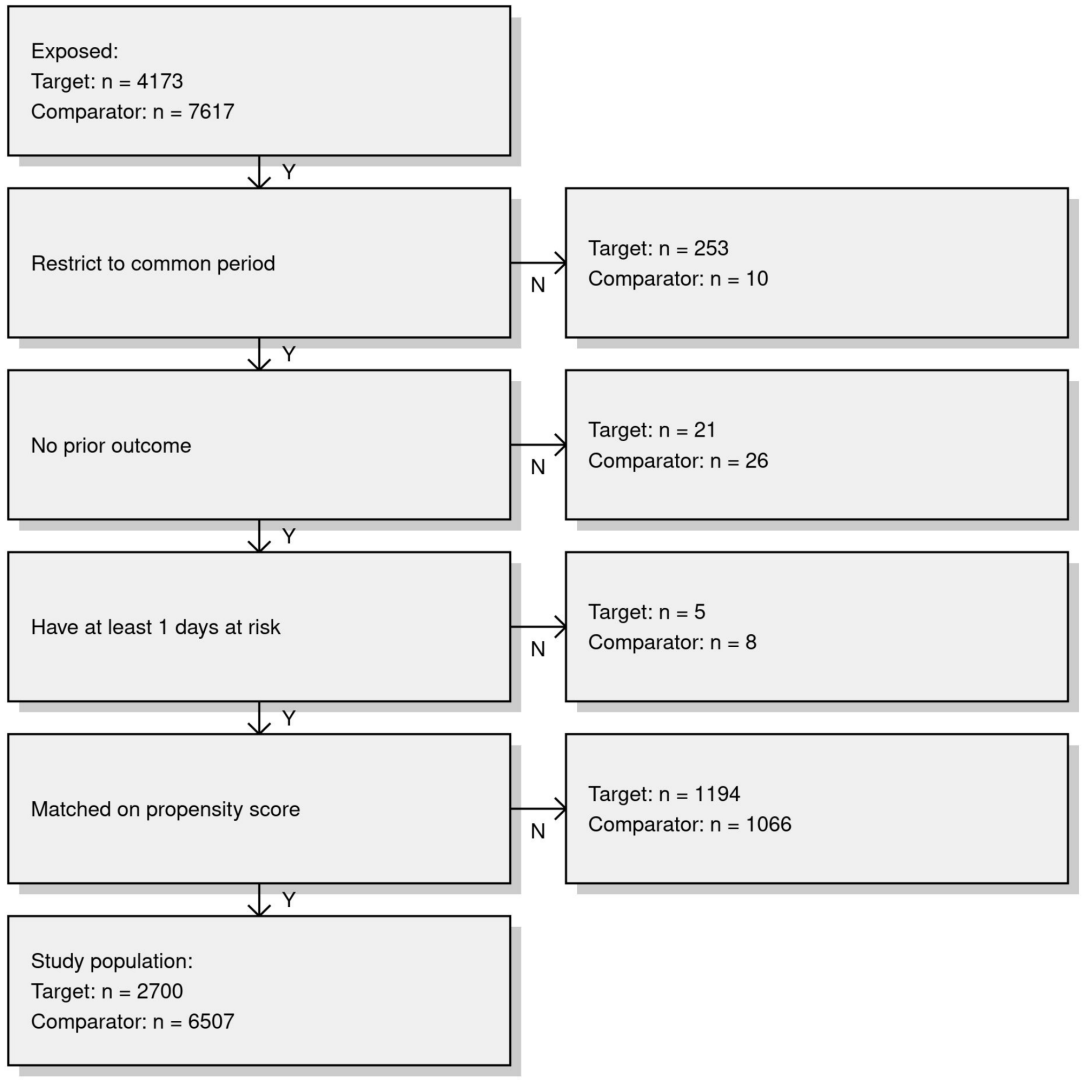
Attrition diagram for rheumatoid arthritis (RA); patient attrition counts and proportions after sequential design choices applied in the Optum^®^ EHR database Key – Target: patients with RA newly exposed to Remicade^®^, Comparator: patients with RA newly exposed to golimumab, certolizumab pegol, ustekinumab, vedolizumab]

Figure 5 reports attrition counts for design restrictions of the primary analysis in Optum^®^ EHR, the one database that passed diagnostics for the comparison in the RA study population. The initial target population was 4,173, which was reduced to 2,700 (65%) after PS matching and other design restrictions were applied.

The target and comparator IRs were 2.93 and 1.13/1k PYs classifying NIU in the exposed populations with RA as uncommon and rare, respectively [62]. Among 2,700 target patients PS matched to 6,507 comparator patients, we observed 5 and 4 events respectively with cHR 1.23 (95% CI 0.14, 10.47). Although this suggests no strong evidence of an increased risk, the low event counts and high imprecision makes it difficult to conclude whether an increased NIU risk exists for Remicade^®^ new users in the RA study population.

##### AS, PsO/PsA

No exposure comparisons in the AS and PsO/PsA study population passed all validity diagnostics, so no NIU safety evidence was generated.

#### Secondary findings

The findings from our secondary analyses were broadly like those of the primary analysis (Fig. 4). Of the 60 secondary analyses, 15 passed all diagnostics and were reviewed (Appendix 7). In the IBD study population under the 1:10 PS matched ITT analysis, we observed a pooled cHR of 0.67 (95% CI 0.43, 1.04). Further, under the 1:1 PS matched ITT analysis, the pooled cHR was 0.66 (95% CI 0.41, 1.06). We observed a pooled cHR of 0.64 (95% CI 0.32, 1.31) in the 1:1 PS matched on-treatment analysis. The narrower CIs of the ITT analysis could suggest more NIU events were observed in the comparator TAR after the on-treatment time, but we recognize this TAR was not our primary analysis for making inference. In the RA study population under the 1:10 PS matched ITT analysis, we observed a pooled cHR 1.09 (95% CI 0.44, 2.71) in the Optum^®^ EHR database. Further, under the 1:1 PS matched ITT analysis the pooled cHR was 1.74 (95% CI 0.55, 5.48) also in Optum^®^ EHR. Regardless of the database, study population, PS matching strategy, or the TAR, we observed no evidence that Remicade^®^ was causally associated with an increased risk for NIU relative to alternative therapies used within the same indication study population. Full details on these clinical finding with full diagnostics results can be found at the **Estimation Diagnostics Explorer**.

## Discussion

We assessed the threats to validity of Remicade^®^ comparative safety effects on NIU using four pre-specified diagnostics. Four of our 20 primary analyses conducted across a network of 5 US observational databases passed all validity diagnostics. Of the 80 primary and secondary analyses, 19 of 80 (24%) passed all diagnostics. That most analyses failed diagnostics suggests that the clinical, scientific, and analytic complexity of the research question applied to heterogenous databases was not easily addressed despite the application of many best practices in the field supported by robust methodological research [63]. This complexity notwithstanding, we identified three databases that passed validity diagnostics in the IBD study population, which facilitated a meta-analysis.

The methodological implementation of the research question was complex for two reasons. First, because Remicade^®^ is indicated for several autoimmune disorders that have primary manifestations such as joint pain and bowel inflammation but can also have secondary manifestations such as NIU. We accounted for potential confounding by indication by designing an active comparator cohort study where the target and comparator exposures were both indicated for the same underlying disease, thereby balancing the baseline risk for NIU. Because NIU risk differs among Remicade® indicated populations [64], we approximated equal baseline NIU risk distribution between target and comparator cohorts by conducting comparisons within non-mutually exclusive study populations (i.e., IBD, PsA/PsO, RA, and AS). Second, Remicade^®^ is an early biologic therapy with five adult indications [65] for which alternative therapies are available. This complexity was reflected in the finding that of 16 analyses × 5 databases = 80 effect estimates, 61 (76%) failed diagnostics and did not produce an estimate we could interpret as causal.

**Empirical equipoise** is a state in which a health care provider would be indifferent to treatment choice given the benefits and risks of competing potential therapies and the known clinical history of a patient. In the context of a comparative study, empirical equipoise is identified as a proportion of patients for whom provider preference for a target or comparator treatment is equivalent. Although we designed our treatment comparisons to be among patients with the same underlying conditions to control for confounding by indication and create more balanced groups, patient characteristics may still have influenced treatment preference. Empirical equipoise was proposed as a feasibility diagnostic to determine whether most (≥ 50%) patients in a comparative effect estimation study would be near equally likely (between 0.3 and 0.7 of the preference score distribution) to be assigned either treatment, conditional on their clinical history [56]. All primary analyses in the PsO/PsA study population failed empirical equipoise at our lower threshold of 35%, which suggests that the multi-drug comparator cohort of [golimumab, certolizumab pegol, guselkumab, risankizumab, tildrakizumab, brodalumab, ixekizumab, secukinumab, or ustekinumab] may not represent suitable exposures for comparison to Remicade^®^ in this population. It is possible that patient heterogeneity among the multi-drug comparator population made equipoise difficult to achieve. Of the primary analyses that failed equipoise, two failed the zero events diagnostic (Amb EMR and Clinformatics^®^) and one failed EASE (Amb EMR). Given that less than 35% of patients were in equipoise for all databases in this study population, it followed that PS matching failed to achieve covariate balance in the one database (Optum EHR^®^) with the largest cohort sizes and most data for LSPS modeling. This finding lends support the use of empirical equipoise as a practical feasibility tool as was its original intent.

In the large IBD study population, we achieved a priori specified **covariate balance** in four of the five databases, although as noted above, covariate balance failure in Clinformatics^®^ was based on one covariate which was unlikely to be a confounder because of a 0.007% baseline prevalence target comparator-difference of ‘Requires Bacillus Calmette-Guerin vaccination.’ Although likely inapplicable in this clinical content, this finding illustrates the utility of using LSPS adjustment methods. The classic directed acyclic graph intended to encode confounding is the following: A → Y and A ← **X** → Y, where A = exposure status, Y = outcome, and **X** = [vector of confounding covariates]. Typically, investigators will select confounders to balance patients of differing exposure status using expert opinion or a screening algorithm [66], where the former method assumes known confounding structure and covariates and the latter relies on analyzing outcome associations before establishing covariate balance [67–69]. An advantage to the LSPS approach is that it assumes no confounding structure, nor does it analyze the outcome in identifying confounders for balancing. Rather, by including all baseline covariates as candidate predictors of exposure status, the LSPS selects and includes highly discriminative covariates into a final parsimonious model and shrinks the coefficients of covariates of low discriminative importance (some to zero, removing them from the final model). A consequence of this approach is that after adjustment with LSPS built with sufficiently large cohorts with rich baseline data, *all* baseline covariates will often be sufficiently balanced, some of which will be confounders. This effectively erases the **X** → A edge, which eliminates observed confounding and requires no untestable confounding structure assumptions. In the smaller RA study populations, cohort size and associated baseline covariate data were sufficiently rich to achieve cohort balance in Optum^®^ EHR. Although covariate imbalance was substantially reduced after LSPS matching in the RA study population in all databases, the diagnostic criterion only passed in Optum^®^ EHR. All comparisons in the AS study population failed covariate balance and four databases in the PsA/PsO study populations failed except for Optum^®^ EHR. In many comparisons covariate imbalance was considerably reduced, which is encouraging, but these analyses did not meet our pre-specified diagnostic criteria to which we strictly adhered. Similarly, in the RA study population, only Optum^®^ EHR passed the covariate balance diagnostic. It is worth noting that the LSPS is a data intensive predictive algorithm that better balances baseline covariates after training on large, high dimensional input data [47]. The Optum^®^ EHR database, in which the AS and PsA/PsO comparisons passed covariate balance, had the largest study populations of the five databases analyzed.

**EASE** is a metric that summarizes the systematic error component of the empirical null distribution fit from negative control outcome effect estimates intended to identify and correct for unobserved confounding. Comparative analyses for all study populations (IBD, PsA/PsO, AS, and RA) failed the EASE diagnostic in the Amb EMR database. Compared to insurance claims databases, electronic health record databases like Amb EMR generally have less observable patient time and it is more inconsistently captured [70]. A consequence of this is that periods of inferred persistent drug exposure are shorter in electronic health records which results in less opportunity to observe outcome events such as negative controls during post exposure TAR periods. As such, we observed relatively few negative control events in the Amb EMR analyses, which resulted in large, statistically unstable EASE values. The lowest EASE value we observed was 0.28 in the IBD analysis from an empirical null distribution computed from 28/86 (33%) observed negative controls. The largest EASE value was 0.98 in the AS study population from an empirical null distribution computed from 4/86 (5%) observed negative controls. This large EASE value was highly influenced by a single large negative control HR. In the IBD, RA, and AS study populations, Remicade^®^ increased the risk of negative controls on average, whereas it reduced the risk in the PsA/PsO study population. These findings suggest that the EASE diagnostic and negative controls-based calibration is sensitive to the patient observable time, drug exposure durations, and the frequency of negative control occurrence during exposure TAR.

Causal studies estimating the effect of an exposure on an outcome do not necessarily rely on **representativeness** and in some cases requiring sample representativeness can be counterproductive to establishing the internal validity on which causal assumptions rely [60]. However, it may be worth reporting to evidence consumers the extent to which patient attrition from observational study design choices impact the constitution of the target study population. In our study, except for the RA study population analysis in the Pharmetrics database (Fig. 2 [row 2, column 4]), the patient restrictions from our study design choices did not substantially alter the composition of the Remicade^®^ target population.

We strove to reduce outcome misclassification by developing a NIU definition by following a novel phenotyping process [39]. In this data driven approach, we specified three candidate computable phenotype algorithms to adhere as closely as possible to a complete physiological, clinical description of NIU. Our algorithms adhered to this clinical description to the extent possible given temporal logic constraints and code availability in standardized medical vocabularies [71]. We then comprehensively characterized the patient cohorts returned by the algorithm in the five databases and mapped the results against the clinical description and determined the extent to which the characteristics reflected the description. Further, we evaluated the candidate definitions by estimating misclassification errors using a probabilistic reference standard, a method designed for flexible and scalable validation [40, 41]. As it relates to comparative effect estimation, our NIU definition had high specificity which will limit bias from misclassification toward HR = 1.

The importance of phenotyping to support observational research is difficult to understate. When rigorously and transparently developed and evaluated, a phenotype definition acts as a reliable, consistent input to any observational analysis intended to study that patient population. Further, when defined against a common data model, the definition is transportable and easily implement across databases, facilitating results interpretation across sources. In short, good phenotyping practices create reusable definitions for use as consistent inputs to support standardized, repeatable, and reproducible evidence generation. For example, since development, our NIU definition was included in the OHDSI community ‘How Often’ initiative (https://github.com/ohdsi-studies/HowOften), a large-scale incidence rate characterization study intended to systematically generate incidence evidence across a large set of conditions across a global, distributed database network [30].

### Attributes of reliable evidence

Attributes of reliable evidence are that it is repeatable, reproducible, replicable, generalizable, robust, and calibrated [71]. Our study is repeatable and reproducible in that investigators with access to the same data, standardized to a version-controlled CDM, should be able to apply our exact analysis and produce an identical result. Repeatable and reproducible evidence implies a publicly available, fully pre-specified protocol including methodological rationale (https://github.com/ohdsi-studies/UveitisSafetyEstimation/tree/master/Documents) and accessible source code to review the analytic implementation process (https://github.com/ohdsi-studies/UveitisSafetyEstimation). Our study is replicable in that we asked the same research question using identical analytic routines against several similar data sources (e.g., multiple US commercial insurance claims databases) which yielded comparable results. Our confidence in the reliability of this evidence is strengthened further by its generalizability, since we observed consistent results across databases of varying content and intent (e.g., insurance claims and EHRs). We subjected our analysis to several sensitivity analyses where we were uncertain of our design specifications which resulted in consistent results, indicating that our results are robust. Lastly, our results are calibrated through our verifying the study design and implementation with design inputs expected to produce known results (i.e., null effects from negative control outcomes). Lastly, and in alignment with the LEGEND principles [28], we specified our study to produce a comprehensive set of effect estimates and we reported them all in the **Estimation evidence explorer** to avoid p-hacking and facilitate fully transparent review, which we encourage. Lastly, the replicability, generalizability, robustness, and calibration evidence attributes can be evaluated in full by reviewing the detailed results of our validity diagnostics, also available in the **Estimation evidence explorer.**

### Clinical implications

NIU can lead to visual impairment and in some cases, blindness [72], thus understanding whether new use of the widely prescribed biologic product Remicade^®^ among large indicated populations may increase the risk of NIU is crucial. NIU is a known secondary manifestation of underlying autoimmune disease, so disambiguating causal risk attributable to confounding by indication rather than autoimmune disease therapy with Remicade^®^ is complex, particularly given that there is also an association between use of certain medications and development of NIU [72]. Through the use of observational data sources available and the causal methods employed, we did not observe evidence of an increased NIU risk attributable to Remicade^®^ in these analyses. We note, importantly, that our analyses were imprecise as shown by the wide CIs, so we cannot confidently rule out the hypothesis of no effect.

These findings must be interpreted within the context of existing literature on similarly indicated TNF inhibitors and their association with NIU. Etanercept, a TNFα and TNFβ inhibitor, is an alternative therapy for PsO/PsA, RA, and AS and has been shown to increases risk of NIU [33, 35]. Adalimumab, another TNFα inhibitor, is another therapy indicated for IBD, PsO/PsA, RA, AS, but also for NIU. Several studies have demonstrated this product is effective in treating and reducing the risk of uveitis [34]. Given the existing evidence on biologic therapies with similar mechanisms of action as Remicade^®^ and their known increased and decreased risks of NIU, we must exercise caution interpreting our finding of no effect given the imprecision of our estimates.

No exposure comparisons in the AS and PsO/PsA study population passed all validity diagnostics, so no NIU safety evidence was generated. This finding itself is useful evidence, however. We have learned that this study design and specification is not supported by these data to reliably answer this NIU causal safety question in the AS and PsO/PsA patient populations. The implications are non-trivial insofar as results from a similar study insufficiently interrogated by rigorous study diagnostics could lead to potentially harmful clinical or policy action.

### Strengths and limitations

This study has several strengths. First, it leveraged data from five large US-based observational health care databases that provided a large, comprehensive sample of commercially insured patients treated with biologics from which we could evaluate an important safety outcome across diverse settings. We used best practices for pharmacoepidemiologic causal estimation by conducting the new user active comparator design [24, 25], using LSPS to adjust for directly and indirectly measured confounders [46], meaningful comparisons based on extensive diagnostics. The integration of negative control outcomes as a diagnostic tool enhances the study’s capacity to identify and assess systematic errors within its design. Furthermore, a meticulous clinical characterization of patients with non-infectious uveitis was undertaken, ensuring the validity of the outcome cohort definition, with the added credibility of an ophthalmologist’s review. The study also performed thorough characterization of exposure within indication populations.

Despite its strengths, we acknowledge several limitations that warrant consideration. Data quality and clinical event misclassification concerns arise in repurposing administrative data and electronic health record data for clinical research. Data quality was assessed during data standardization to the OMOP CDM [31, 73] and through explicit data quality assessment [74] and deemed sufficient for clinical research purposes. For the key study population and outcome variables we followed rigorous phenotype development and evaluation processes [39–41], that have been applied elsewhere in the observational research literature [75, 76].

The clinical community and literature in the field acknowledges that disease severity and reasons for treatment switching to biologics are important patient characteristics that may confound these exposure-outcome relationships. These characteristics are poorly recorded or absent from the data we used, but we sought mitigate this limitation by two methods. We employed LSPS methodology which balances all observed and indirectly measured baseline covariates. Also, we employed empirical calibration that measures and calibrates effect estimates for residual bias after other bias mitigation strategies like PS matching were conducted.

Notably, while we quantified outcome misclassification during phenotype development and evaluation, we did not correct effect estimates for this source of bias. Further we did not calculate misclassification errors by exposure status. However, if we assume the high specificity is non-differential to exposure status, this suggests the effect estimate would be minimally biased from outcome misclassification [42, 77]. When outcome specificity depends on exposure status, bias could be considerable, especially in low outcome prevalence settings [78].

Also, attrition to the after matching cohort by excluding non-PS matched patients poses a potential threat to external validity, raising concerns about the generalizability of results to Remicade^®^-exposed populations as initially defined, as well as power to detect safety effects. Due to the attrition, we performed characteristic comparisons between the initial and after-matching target cohorts that demonstrated similarity suggesting generalizability, although this finding is supportive of our ATO estimate targeting the ATT. Alternatively, ATT weights would target the ATT without needing to demonstrate representativeness, given that no treated patients are excluded using this method. However, methods evaluation work using average treatment effect (ATE) weights demonstrated poor coverage, increase risk on negative controls [63] and imprecise estimates [79] likely because of the presence of extreme weights. Other weighting methods that are less prone to influence of extreme weights, such as fine stratification weights [80], would be a useful direction for future research.

Additionally, in this study, a condition for causal inference, and in turn for the results from a database to be included in the meta-analysis, was that both target and comparator cohorts each had at least one event observed during their respective TARs. Exploration of novel meta-analysis methods capable of relaxing this constraint is recommended [81]. For example, a Bayesian random-effects meta-analysis that uses non-normal likelihood approximations can reduce bias and increase precision of the treatment effect in future multi-database studies facing low and zero event counts.

Lastly, our use of LSPS was intended to balance all observed baseline covariates, of which a subset are likely confounders that if imbalanced would lead to biased effect estimates. However, this method requires that many baseline covariates that are not confounders to be similarly balanced given their likely association with unobserved confounders [46]. It is possible that some analyses that failed the covariate balance diagnostic were from imbalanced covariates that would not otherwise bias the effect estimate.

## Conclusion

We conducted a comparative cohort across five data sources and four indications intended to estimate the causal association between Remicade^®^ and NIU. We applied best practices methods for PS adjustment and unobserved confounding control. Three quarters (76%) of the total primary and secondary analyses failed to pass the pre-specified diagnostic thresholds, so we did not review the effect estimates because we could not interpret these results as causally valid. In our primary analyses that passed validity diagnostics, we failed to reject the null hypothesis of no effect and conclude that we observed no strong evidence of an increased risk of NIU among new users of Remicade^®^ in the IBD and RA study populations relative to their respective comparator exposure cohorts. We observed consistent clinical results in or secondary analyses that passed diagnostics We note that our final estimates were imprecise given the rarity of NIU occurrence, but as data accrue, we could foreseeably re-execute our repeatable, reproducible study to potentially increase estimate precision.

Generating reliable causal evidence from an observational study is possible and the study’s quality is improved by explicitly acknowledging its causal intent [82]. We assert that the causal evidence in this case study and other similar observational, comparative effect estimation studies (e.g., [19, 20, 83–88]) is strengthened by the application of these validity diagnostics. We further believe that the reliability of observational causal evidence can only be confirmed through the transparent, rigorous application of these diagnostics. We believe that the credibility of casual inference in observational data depends on it. We share the conviction of regulatory authorities that properly conducted observational studies can produce comparative safety and effectiveness evidence of sufficient quality to complement evidence from RCTs and inform regulatory decisions [8, 14–16, 89, 90]. The limitations of RCTs are well-established [91]. This presents an opportunity for observational researchers to fill evidentiary gaps where RCT evidence is infeasible, unethical, inapplicable, or otherwise unavailable.

## Electronic supplementary material

Below is the link to the electronic supplementary material.

Supplementary Material 1

## Data Availability

Databases used in this study are available via license are available for purchase from Merative^®^ (CCAE), Optum^®^ (Clinformatics^®^ and Optum^®^ EHR), and IQVIA (Pharmetrics and Amb EMR). Aggregated (i.e., no person-level data) results that are the basis of the study findings are publicly available at: (1) https://github.com/OHDSI/ShinyDeploy/tree/master/UveitisSafetyEstimation/data, (2) https://github.com/OHDSI/uvetis_safety.

## Footnotes

1 Note that in most uses related to research data, the term ‘real-world’ is interchangeable with the term ‘observational’. For example, the terms RWD and RWE are equivalent to observational data and observational evidence, respectively. The term ‘observational’ will be used for the remainder of this document.

2 Remicade^®^ should be administered in combination with methotrexate for the treatment of rheumatoid arthritis.

3 Janssen Biotech, Inc. (2021). Remicade^®^ (infliximab) [prescribing information]. Food and Drug Administration https://www.accessdata.fda.gov/drugsatfda_docs/label/2020/103772s5389s5391s5394lbl.pdf.
