# Supplementary material for: The necessity of validity diagnostics when drawing causal inferences from observational data: lessons from a multi-database evaluation of the risk of non-infectious uveitis among patients exposed to Remicade®
Source: BMC Med Res Methodol. 2024 Dec 27;24:322. doi: 10.1186/s12874-024-02428-7 (PMC11681708; doi:10.1186/s12874-024-02428-7)
Supplement: Supplementary file 1 — Supplementary Material 1 [file 12874_2024_2428_MOESM1_ESM.pdf]

Supplementary Appendix to:

The necessity of validity diagnostics when drawing causal inferences from observational data: lessons from a multi-database evaluation of the risk of non-infectious uveitis among patients exposed to Remicade®

James Weaver[1], Erica A Voss[1], Guy Cafri[2], Kathleen Beyrau[3], Michelle Nashleanas[3], Robert Suruki[1]

[1]Janssen Research & Development, LLC, Global Epidemiology Organization, [2]Johnson & Johnson MedTech Epidemiology and Real-World Data Sciences, [3]Johnson & Johnson Global Medical Safety

Appendix 1: Database Details

CCAE

Merative™ MarketScan® Commercial Database (CCAE)

Database Start Date 2000-01-01
Database End Date 2022-01-31

The Merative™ MarketScan® Research Databases contain individual-level, de-identified, healthcare claims information from employers, health plans, hospitals, and Medicare and Medicaid programs. Since their creation in the early 1990s, the MarketScan Databases have grown into one of the largest collections of de-identified patient-level data in the United States. These databases reflect real-world treatment patterns and costs by tracking millions of patients as they travel through the healthcare system, offering detailed information about all aspects of care. Data about individual patients are integrated from all providers of care, maintaining healthcare utilization and cost record connections at the patient level. Used primarily for research, these databases are fully compliant with U.S. privacy laws and regulations (i.e., HIPAA). Research using MarketScan® data has been widely published in peer-reviewed medical and health services journals. MarketScan® is a registered trademark of Merative™ Corporation in the United States, other countries, or both.

Merative™ MarketScan® Commercial Database are data including health insurance claims across the continuum of care (e.g., inpatient, outpatient, outpatient pharmacy, carve-out behavioral healthcare) as well as enrollment data from large employers and health plans across the United States who provide private healthcare coverage for millions of employees, their spouses, and dependents. These administrative claims database includes a variety of fee-for-service, preferred provider organizations, and capitated health plans.

MarketScan is a registered trademark of Merative™ Corporation in the United States, other countries, or both

Clinformatics®

Optum© De-Identified Clinformatics® Data Mart Database (Clinformatics®)

Database Start Date 2000-05-01
Database End Date 2021-12-31

Optum's Clinformatics® Data Mart is derived from a database of administrative health claims for members of large commercial and Medicare Advantage health plans. The database includes

approximately 17-19 million annual covered lives, for a total of over 65 million unique lives over a 12-year period (1/2007 through 12/2019). Clinformatics® Data Mart is statistically de-identified under the Expert Determination method consistent with HIPAA and managed according to Optum® customer data use agreements. Administrative claims submitted for payment by providers and pharmacies are verified, adjudicated and de-identified prior to inclusion. This data, including patient-level enrollment information, is derived from claims submitted for all medical and pharmacy health care services with information related to healthcare costs and resource utilization. The population is geographically diverse, spanning all 50 states. Optum Clinformatics® Data Mart Date of Death (Optum DOD) also provides date of death (month and year only) for members with both medical and pharmacy coverage from the Social Security Death Master File (however after 2011 reporting frequency changed due to changes in reporting requirements) and location information for patients is at the US state level.

Pharmetrics

IQVIA Adjudicated Health Plan Claims Data (IQVIA_PHARMETRICS_PLUS)

Database Start Date	2013-01-01
Database End Date	2021-11-30

The IQVIA Adjudicated Health Plan Claims Data (formerly Pharmetrics Plus) – a United States of America (US) database comprised of fully adjudicated health plan claims data and enrollment information for commercial individuals. The information is comprised of over 70 contributing health plans and self-insured employer groups throughout the US for over more than 140 million unique enrollees since 2006. This anonymous, patient-centric database includes all medical and pharmacy claims data (costs and descriptive services). Claims represent payments to providers for services rendered to covered health plan individuals. The data also includes patient-level enrollment which is a record of demographic variables including eligibility status (year of birth, gender, US Census region, eligibility by month). The enrollee population in the database is generally representative of the less than 65 years of age, commercially insured population with a subset of Commercial Medicare and Medicaid in the US with respect to both age and gender. The average length of enrollment is 39 months, and 47 million patients have 3 or more years of continuous enrollment (medical and pharmacy coverage). Each contributing plan's data undergoes rigorous data quality review by IQVIA prior to its addition to the IQVIA Adjudicated Health Plan Claims - US database.

Optum® EHR

Optum® de-identified Electronic Health Record Dataset

Database Start Date	2007-01-01
Database End Date	2022-03-01

Optum's longitudinal EHR repository is derived from dozens of healthcare provider organizations in the United States, that include more 57 contributing sources and 111K sites of care and includes approximately 107 million patients. The data is certified as de-identified by an independent statistical expert following HIPAA statistical de-identification rules and managed according to Optum® customer data use agreements. Clinical, claims and other medical administrative data is obtained from both Inpatient and Ambulatory electronic health records (EHRs), practice management systems and numerous other internal systems. Information is processed, normalized, and standardized across the

continuum of care from both acute inpatient stays and outpatient visits. Optum® data elements include demographics, medications prescribed and administered, immunizations, allergies, lab results (including microbiology), vital signs and other observable measurements, clinical and inpatient stay administrative data and coded diagnoses and procedures. In addition, Optum® uses natural language processing (NLP) computing technology to transform critical facts from physician notes into usable datasets. The NLP data provides detailed information regarding signs and symptoms, family history, disease related scores (i.e., RAPID3 for RA, or CHADS2 for stroke risk), genetic testing, medication changes, and physician rationale behind prescribing decisions that might never be recorded in the EHR.

Amb EMR

IQVIA Ambulatory Electronic Medical Records

Database Start Date	1995-01-01
Database End Date	2021-12-24

The IQVIA Ambulatory EMR asset is comprised of approximately 90+ million patient records with a face-to-face physician interaction since 2006 and are sourced from an "opt-in" provider research network.

The aggregated database comprises records collected by over 100,000 physicians from large practices and physician networks located in all 50 US states. Approximately 40% of the contributing physicians are primary care practitioners and the remaining are specialists.

Key information collected includes:

- Patient age/ gender / race / 3-digit ZIP/longitudinal tracking
- Vitals (e.g., blood pressure, heart rate, weight, height)
- Risk factors (e.g., smoking, alcohol), co morbidities, medical history
- Lab tests performed and results
- Allergies and vaccine details
- Diagnoses (ICD-9, ICD-10)
- Prescription drugs prescribed, administered (strength, form, quantity, frequency, days' supply, refills, DAW, start and stop dates)
- Procedures performed/other treatments (CPT)
- SNOMED-CT codes
- Recording of patient care episodes (encounters, visits, appointments, correspondence, etc.)

Incremental data contributions are processed monthly. Users will typically see a 45-day lag before new information is added to the database.

The information collected allows linking key clinical variables such as lab values and blood pressure to therapeutic outcomes and to prescriptions / diagnosis / hospital metrics and connecting patient vitals, health behaviors and risk factors to diagnosis and treatment.

Appendix 2: Study Populations

Cohort Concept Sets

This file can be used to find the standardized OMOP concepts, from the OMOP Vocabulary, which are used in the cohort definitions below. The attached file has a column titled "CONCEPT_SET_NAME", that can be used to locate the concepts sets mentioned. Concept sets will be surrounded by single quotes in the cohort definitions below. For example, to find the 'Crohn's disease' concept set, filter to that condition in the "CONCEPT_SET_NAME" field.

https://github.com/ohdsi-studies/UveitisSafetyEstimation/tree/master/Documents

Indication Cohorts

Crohn's Disease & Ulcerative Colitis

Cohort Entry Events

People enter the cohort when observing any of the following:

1. condition occurrences of 'Crohn's disease'.
2. condition occurrences of 'Ulcerative colitis'.

Limit cohort entry events to the earliest event per person.

Cohort Exit

The person exits the cohort at the end of continuous observation.

Cohort Eras

Entry events will be combined into cohort eras if they are within 0 days of each other.

Ankylosing Spondylitis

Cohort Entry Events

People enter the cohort when observing any of the following:

- condition occurrence of 'Ankylosing Spondylitis' for the first time in the person's history.

Limit cohort entry events to the earliest event per person.

Cohort Exit

The person exits the cohort at the end of continuous observation.

Cohort Eras

Entry events will be combined into cohort eras if they are within 0 days of each other.

Moderate to severe plaque psoriasis & Psoriatic arthritis

Cohort Entry Events

People enter the cohort when observing any of the following:

1. condition occurrences of 'Plaque psoriasis'.
2. condition occurrences of 'Psoriatic arthritis (exclude mutilans)'.
3. condition occurrences of 'Arthropathy'; having at least 1 condition occurrence of 'Psoriasis', starting between 60 days before and 60 days after 'Arthropathy' start date.

Limit cohort entry events to the earliest event per person.

Cohort Exit

The person exits the cohort at the end of continuous observation.

Cohort Eras

Entry events will be combined into cohort eras if they are within 0 days of each other.

Rheumatoid Arthritis

Cohort Entry Events

People enter the cohort when observing any of the following:

1. condition occurrence of 'Rheumatoid arthritis' for the first time in the person's history.
2. observation of 'Rheumatoid arthritis' for the first time in the person's history.

Limit cohort entry events to the earliest event per person.

Cohort Exit

The person exits the cohort at the end of continuous observation.

Cohort Eras

Entry events will be combined into cohort eras if they are within 0 days of each other.

Target Cohorts

Remicade with IBD

Cohort Entry Events

People with continuous observation of 365 days before event may enter the cohort when observing any of the following:

1. drug exposure of '[964] Infliximab (Remicade) plus source code' for the first time in the person's history, who are >= 18 years old; starting on or after August 24, 1998; having no drug exposures of '[964] Infliximab (all brands)', starting any time prior to '[964] Infliximab (Remicade) plus source code' start date.

Limit cohort entry events to the earliest event per person.

Inclusion Criteria

1. No prior other TNFαi

Entry events with all of the following criteria:

   a. having no drug exposures of '[964] adalimumab', starting anytime on or before cohort entry start date; allow events outside observation period.
   b. having no drug exposures of '[964] certolizumab pegol', starting anytime on or before cohort entry start date; allow events outside observation period.
   c. having no drug exposures of '[964] etanercept', starting anytime on or before cohort entry start date; allow events outside observation period.
   d. having no drug exposures of '[964] golimumab', starting anytime on or before cohort entry start date; allow events outside observation period.

2. No prior vedolizumab or natalizumab

Entry events with all of the following criteria:

   a. having no drug exposures of '[964] vedolizumab', starting anytime on or before cohort entry start date; allow events outside observation period.
   b. having no drug exposures of '[964] natalizumab', starting anytime on or before cohort entry start date; allow events outside observation period.

3. No prior IL

Entry events having no drug exposures of '[EPI_964] Interleukin inhibitors', starting anytime on or before cohort entry start date; allow events outside observation period.

4. Prior IBD

Entry events will have all of the following criteria:

   a. having at least one condition occurrence of 'Crohn’s Disease or Ulcerative Colitis', starting anytime on or before cohort entry start date; allow events outside observation period

Cohort Exit

The cohort end date will be based on a continuous exposure to '[964] Infliximab (Remicade) plus source code': allowing 90 days between exposures, adding 90 days after exposure ends, and forcing drug exposure days supply to: 1 day. The person exits the cohort when encountering any of the following events:

1. drug exposures of '[964] adalimumab'.
2. drug exposures of '[964] golimumab'.
3. drug exposures of '[964] certolizumab pegol'.
4. drug exposures of '[964] etanercept'.
5. drug exposures of '[EPI_964] Interleukin inhibitors.
6. drug exposures of '[964] vedolizumab'.
7. drug exposures of '[964] natalizumab'.

Cohort Eras

Entry events will be combined into cohort eras if they are within 0 days of each other.

Remicade with AS

Cohort Entry Events

People with continuous observation of 365 days before event may enter the cohort when observing any of the following:

1. drug exposure of '[964] Infliximab (Remicade) plus source code' for the first time in the person's history, who are >= 18 years old; starting on or after December 17, 2004; having no drug exposures of '[964] Infliximab (all brands)', starting any time prior to '[964] Infliximab (Remicade) plus source code' start date.

Limit cohort entry events to the earliest event per person.

Inclusion Criteria

1. No prior other TNFαi

   Entry events with all of the following criteria:

   a. having no drug exposures of '[964] adalimumab', starting anytime on or before cohort entry start date; allow events outside observation period.
   b. having no drug exposures of '[964] certolizumab pegol', starting anytime on or before cohort entry start date; allow events outside observation period.
   c. having no drug exposures of '[964] etanercept', starting anytime on or before cohort entry start date; allow events outside observation period.
   d. having no drug exposures of '[964] golimumab', starting anytime on or before cohort entry start date; allow events outside observation period.
2. No prior IL

   Entry events having no drug exposures of '[EPI_964] Interleukin inhibitors', starting anytime on or before cohort entry start date; allow events outside observation period.

3. Prior AS

   Entry events will have all of the following criteria:

   a. having at least one condition occurrence of 'Ankylosing Spondylitis', starting anytime on or before cohort entry start date; allow events outside observation period

Cohort Exit

The cohort end date will be based on a continuous exposure to '[964] Infliximab (Remicade) plus source code': allowing 90 days between exposures, adding 90 days after exposure ends, and forcing drug exposure days supply to: 1 day. The person exits the cohort when encountering any of the following events:

1. drug exposures of '[964] adalimumab'.

2. drug exposures of '[964] golimumab'.
3. drug exposures of '[964] certolizumab pegol'.
4. drug exposures of '[964] etanercept'.
5. drug exposures of '[EPI_964] Interleukin inhibitors.

Cohort Eras

Entry events will be combined into cohort eras if they are within 0 days of each other.

Remicade with PsO/PsA

Cohort Entry Events

People with continuous observation of 365 days before event may enter the cohort when observing any of the following:

1. drug exposure of '[964] Infliximab (Remicade) plus source code' for the first time in the person's history, who are >= 18 years old; starting on or after May 13, 2005; having no drug exposures of '[964] Infliximab (all brands)', starting any time prior to '[964] Infliximab (Remicade) plus source code' start date.

Limit cohort entry events to the earliest event per person.

Inclusion Criteria

1. No prior other TNFαi

   Entry events with all of the following criteria:

   a. having no drug exposures of '[964] adalimumab', starting anytime on or before cohort entry start date; allow events outside observation period.
   b. having no drug exposures of '[964] certolizumab pegol', starting anytime on or before cohort entry start date; allow events outside observation period.
   c. having no drug exposures of '[964] etanercept', starting anytime on or before cohort entry start date; allow events outside observation period.
   d. having no drug exposures of '[964] golimumab', starting anytime on or before cohort entry start date; allow events outside observation period.

   2. No prior IL

   Entry events having no drug exposures of '[EPI_964] Interleukin inhibitors', starting anytime on or before cohort entry start date; allow events outside observation period.

5. Prior PsO/PsA

   Entry events will have all of the following criteria:

   a. having at least one condition occurrence of 'Plaque Psoriasis or Psoriatic Arthritis', starting anytime on or before cohort entry start date; allow events outside observation period

Cohort Exit

The cohort end date will be based on a continuous exposure to '[964] Infliximab (Remicade) plus source code': allowing 90 days between exposures, adding 90 days after exposure ends, and forcing drug exposure days supply to: 1 day. The person exits the cohort when encountering any of the following events:

1. drug exposures of '[964] adalimumab'.
2. drug exposures of '[964] golimumab'.
3. drug exposures of '[964] certolizumab pegol'.
4. drug exposures of '[964] etanercept'.
5. drug exposures of '[EPI_964] Interleukin inhibitors.

Cohort Eras

Entry events will be combined into cohort eras if they are within 0 days of each other.

Remicade and methotrexate with RA

Cohort Entry Events

People with continuous observation of 365 days before event may enter the cohort when observing any of the following:

1. drug exposure of '[964] Infliximab (Remicade) plus source code' for the first time in the person's history, who are >= 18 years old; starting on or after November 10, 1999; with all of the following criteria:
    a. having no drug exposures of '[964] Infliximab (all brands)', starting any time prior to '[964] Infliximab (Remicade) plus source code' start date.
    b. having at least 1 drug exposure of '[964] methotrexate', starting between 30 days before and 30 days after '[964] Infliximab (Remicade) plus source code' start date.

Limit cohort entry events to the earliest event per person.

Inclusion Criteria

1. No prior other TNFαi

    Entry events with all of the following criteria:

    1. having no drug exposures of '[964] adalimumab', starting anytime on or before cohort entry start date; allow events outside observation period.
    2. having no drug exposures of '[964] certolizumab pegol', starting anytime on or before cohort entry start date; allow events outside observation period.
    3. having no drug exposures of '[964] etanercept', starting anytime on or before cohort entry start date; allow events outside observation period.
    4. having no drug exposures of '[964] golimumab', starting anytime on or before cohort entry start date; allow events outside observation period.
2. No prior abatacept

Entry events having no drug exposures of '[964] abatacept', starting anytime on or before cohort entry start date; allow events outside observation period.

3. No prior IL

Entry events having no drug exposures of '[EPI_964] Interleukin inhibitors', starting anytime on or before cohort entry start date; allow events outside observation period.

4. Prior RA

Entry events will have all of the following criteria:

a. having at least one condition occurrence of 'Rheumatoid Arthritis', starting anytime on or before cohort entry start date; allow events outside observation period

Cohort Exit

The cohort end date will be based on a continuous exposure to '[964] Infliximab (Remicade) plus source code': allowing 90 days between exposures, adding 90 days after exposure ends, and forcing drug exposure days supply to: 1 day. The person exits the cohort when encountering any of the following events:

1. drug exposures of '[964] adalimumab'.
2. drug exposures of '[964] etanercept'.
3. drug exposures of '[964] golimumab'.
4. drug exposures of '[964] certolizumab pegol'.
5. drug exposures of '[EPI_964] Interleukin inhibitors.
6. drug exposures of '[964] abatacept'.

Cohort Eras

Entry events will be combined into cohort eras if they are within 0 days of each other.

Comparator Cohorts

golimumab, certolizumab pegol, ustekinumab, vedolizumab with IBD

Cohort Entry Events

People with continuous observation of 365 days before event may enter the cohort when observing any of the following:

1. drug exposure of '[964] golimumab, certolizumab pegol, ustekinumab, vedolizumab' for the first time in the person's history, who are >= 18 years old; starting on or after April 22, 2008.

Limit cohort entry events to the earliest event per person.

Inclusion Criteria

1. No prior other TNFαi

Entry events with all of the following criteria:

a. having no drug exposures of '[964] Infliximab (all brands)', starting anytime on or before cohort entry start date; allow events outside observation period.
b. having no drug exposures of '[964] adalimumab', starting anytime on or before cohort entry start date; allow events outside observation period.
c. having no drug exposures of '[964] etanercept', starting anytime on or before cohort entry start date; allow events outside observation period.

2. No prior natalizumab

Entry events having no drug exposures of '[964] natalizumab', starting anytime on or before cohort entry start date; allow events outside observation period.

3. No prior other IL

Entry events having no drug exposures of '[EPI_964] Interleukin inhibitors', starting anytime on or before cohort entry start date; allow events outside observation period.

4. Prior IBD

Entry events will have all of the following criteria:

a. having at least one condition occurrence of 'Crohn’s Disease or Ulcerative Colitis', starting anytime on or before cohort entry start date; allow events outside observation period

Cohort Exit

The cohort end date will be based on a continuous exposure to '[964] golimumab, certolizumab pegol, ustekinumab, vedolizumab': allowing 90 days between exposures, adding 90 days after exposure ends, and forcing drug exposure days supply to: 1 day. The person exits the cohort when encountering any of the following events:

1. drug exposures of '[964] Infliximab (all brands)'.
2. drug exposures of '[964] adalimumab'.
3. drug exposures of '[964] etanercept'.
4. drug exposures of '[EPI_964] Interleukin inhibitors.
5. drug exposures of '[964] natalizumab'.

Cohort Eras

Entry events will be combined into cohort eras if they are within 0 days of each other.

certolizumab pegol, golimumab, ixekizumab, secukinumab with AS

Cohort Entry Events

People with continuous observation of 365 days before event may enter the cohort when observing any of the following:

1. drug exposure of '[964] certolizumab pegol, golimumab, ixekizumab, secukinumab' for the first time in the person's history, who are >= 18 years old; starting on or after April 24, 2009.

Limit cohort entry events to the earliest event per person.

Inclusion Criteria

1. No prior other TNFαi

   Entry events with all of the following criteria:

   1. having no drug exposures of '[964] Infliximab (all brands)', starting anytime on or before cohort entry start date; allow events outside observation period.
   2. having no drug exposures of '[964] adalimumab', starting anytime on or before cohort entry start date; allow events outside observation period.
   3. having no drug exposures of '[964] etanercept', starting anytime on or before cohort entry start date; allow events outside observation period.
2. No prior other IL

   Entry events having no drug exposures of '[EPI_964] Interleukin inhibitors', starting anytime on or before cohort entry start date; allow events outside observation period.

3. Prior AS

   Entry events will have all of the following criteria:

   a. having at least one condition occurrence of 'Ankylosing Spondylitis', starting anytime on or before cohort entry start date; allow events outside observation period

Cohort Exit

The cohort end date will be based on a continuous exposure to '[964] certolizumab pegol, golimumab, ixekizumab, secukinumab': allowing 90 days between exposures, adding 90 days after exposure ends, and forcing drug exposure days supply to: 1 day. The person exits the cohort when encountering any of the following events:

1. drug exposures of '[964] Infliximab (all brands)'.
2. drug exposures of '[964] adalimumab'.
3. drug exposures of '[EPI_964] Interleukin inhibitors.
4. drug exposures of '[964] etanercept'.

Cohort Eras

Entry events will be combined into cohort eras if they are within 0 days of each other.

golimumab, certolizumab pegol, guselkumab, risankizumab, tildrakizumab, brodalumab, ixekizumab, secukinumab, or ustekinumab with PSO/PsA

Cohort Entry Events

People with continuous observation of 365 days before event may enter the cohort when observing any of the following:

1. drug exposure of '[964] golim, certoliz, guselk, risankiz, tildrakiz, brodal, ixekiz, secukin, ustekin' for the first time in the person's history, who are >= 18 years old; starting on or after April 24, 2009.

Limit cohort entry events to the earliest event per person.

Inclusion Criteria

1. 1. No prior other TNFαi

   Entry events with all of the following criteria:

   1. having no drug exposures of '[964] Infliximab (all brands)', starting anytime on or before cohort entry start date; allow events outside observation period.
   2. having no drug exposures of '[964] adalimumab', starting anytime on or before cohort entry start date; allow events outside observation period.
   3. having no drug exposures of '[964] etanercept', starting anytime on or before cohort entry start date; allow events outside observation period.
2. No prior other IL

   Entry events having no drug exposures of '[EPI_964] Interleukin inhibitors', starting anytime on or before cohort entry start date; allow events outside observation period.

3. Prior PsO/PsA

   Entry events will have all of the following criteria:

   a. having at least one condition occurrence of 'Plaque Psoriasis or Psoriatic Arthritis', starting anytime on or before cohort entry start date; allow events outside observation period

Cohort Exit

The cohort end date will be based on a continuous exposure to '[964] golim, certoliz, guselk, risankiz, tildrakiz, brodal, ixekiz, secukin, ustekin': allowing 90 days between exposures, adding 90 days after exposure ends, and forcing drug exposure days supply to: 1 day. The person exits the cohort when encountering any of the following events:

1. drug exposures of '[964] Infliximab (all brands)'.
2. drug exposures of '[964] adalimumab'.
3. drug exposures of '[EPI_964] Interleukin inhibitors.
4. drug exposures of '[964] etanercept'.

Cohort Eras

Entry events will be combined into cohort eras if they are within 0 days of each other.

certolizumab pegol, tocilizumab with RA

Cohort Entry Events

People with continuous observation of 365 days before event may enter the cohort when observing any of the following:

1. drug exposure of '[964] certolizumab pegol, tocilizumab' for the first time in the person's history, who are >= 18 years old; starting on or after May 13, 2009.

Limit cohort entry events to the earliest event per person.

Inclusion Criteria

1. 1. No prior other TNFαi

   Entry events with all of the following criteria:

   1. having no drug exposures of '[964] Infliximab (all brands)', starting anytime on or before cohort entry start date; allow events outside observation period.
   2. having no drug exposures of '[964] etanercept', starting anytime on or before cohort entry start date; allow events outside observation period.
   3. having no drug exposures of '[964] adalimumab', starting anytime on or before cohort entry start date; allow events outside observation period.
   4. having no drug exposures of '[964] golimumab', starting anytime on or before cohort entry start date; allow events outside observation period.
2. No prior abatacept

   Entry events having no drug exposures of '[964] abatacept', starting anytime on or before cohort entry start date; allow events outside observation period.

3. No prior other IL

   Entry events having no drug exposures of '[EPI_964] Interleukin inhibitors', starting anytime on or before cohort entry start date; allow events outside observation period.

4. Prior RA

   Entry events will have all of the following criteria:

a. having at least one condition occurrence of 'Rheumatoid Arthritis', starting anytime on or before cohort entry start date; allow events outside observation period

Cohort Exit

The cohort end date will be based on a continuous exposure to '[964] certolizumab pegol, tocilizumab': allowing 90 days between exposures, adding 90 days after exposure ends, and forcing drug exposure days supply to: 1 day. The person exits the cohort when encountering any of the following events:

1. drug exposures of '[964] Infliximab (all brands)'.
2. drug exposures of '[964] adalimumab'.
3. drug exposures of '[964] golimumab'.
4. drug exposures of '[EPI_964] Interleukin inhibitors.
5. drug exposures of '[964] abatacept'.
6. drug exposures of '[964] etanercept'.

Outcome Cohort

Non-infectious uveitis or iridocyclitis

(See Appendix 3 for NIU phenotype development and evaluation)

Cohort Entry Events

People enter the cohort when observing any of the following:

1. condition occurrence of 'Non-infectious uveitis or iridocyclitis' for the first time in the person's history; having at least 1 condition occurrence of 'Non-infectious uveitis or iridocyclitis', starting between 31 days after and 365 days after 'Non-infectious uveitis or iridocyclitis' start date.
2. condition occurrences of 'Non-infectious uveitis or iridocyclitis'; having at least 1 visit occurrence of any visit, starting anytime on or before 'Non-infectious uveitis or iridocyclitis' start date and ending between 0 days after and all days after 'Non-infectious uveitis or iridocyclitis' start date; a provider specialty that is: "ophthalmology", "retina ophthalmology", "glaucoma ophthalmology", "pediatric ophthalmology and strabismus", "cornea and external ophthalmology", "pediatric ophthalmology", "neuro-ophthalmology", "uveitis and ocular inflammatory disease ophthalmology" or "orthoptics".

Limit cohort entry events to the earliest event per person.

Cohort Exit

The person exits the cohort at the end of continuous observation.

Cohort Eras

Entry events will be combined into cohort eras if they are within 0 days of each other.

Appendix 3: Non-infectious uveitis phenotype evaluation

Clinical Description

Overview: Non-infectious uveitis and iridocyclitis, often referred to collectively as non-infectious anterior uveitis, are inflammatory eye conditions affecting the uvea, specifically the iris (iritis) and the ciliary body (cyclitis) in the absence of infection. This work refers to this condition as non-infectious uveitis or NIU. The uvea is the middle portion of the eye that extends from the iris, ciliary body to the choroid. These conditions are characterized by inflammation within the anterior segment of the eye and can lead to various ocular complications if left untreated. NIU are considered autoimmune disorders, primarily affecting adults but can occur at any age. Disease etiology includes underlying medical condition risk factors such as ulcerative colitis, and Behcet’s disease.

Synonyms include anterior uveitis, non-granulomatous uveitis, or autoimmune uveitis.

Presentation: Presentation includes nonspecific and variable symptoms in the eye such as visual loss, pain, or redness. On examination there may be signs of leukocytes in the eye chamber. Signs of infection are absent.

Patients with NIU may present with the following non-specific and variable signs and symptoms:

1. *Eye Pain:* Typically described as a dull, aching pain in the affected eye.
2. *Redness (Conjunctival Injection):* Conjunctival vessels become dilated, resulting in a red appearance of the eye.
3. *Photophobia:* Increased sensitivity to light, causing discomfort in well-lit environments.
4. *Blurred Vision:* Impaired vision due to the inflammation and potential complications.
5. *Tearing:* Excessive tearing may occur.
6. *Miosis:* Pupillary constriction.
7. *Floaters:* Patients may notice floaters or spots in their vision.

Diagnostics Evaluation: Assessment requires ophthalmological evaluation of the anterior, middle, and posterior eye chambers.

The diagnosis of NIU is primarily clinical and may involve:

1. *Slit-lamp Examination:* To assess the degree of inflammation and identify specific characteristics such as cells in the anterior chamber (aqueous flare).
2. *Visual Acuity Testing:* To evaluate the impact of inflammation on vision.
3. *Tonometry:* To measure intraocular pressure, as elevated pressure can indicate complications.
4. *Fundoscopy:* To rule out posterior segment involvement.
5. *Laboratory Tests:* Such as complete blood count (CBC), erythrocyte sedimentation rate (ESR), and C-reactive protein (CRP) to assess for underlying systemic causes.

Additionally, ancillary tests such as fluorescein angiography and optical coherence tomography (OCT) may be used to evaluate the extent of inflammation and complications in some cases.

Therapy Plan: Non-infectious uveitis should be managed with urgency to prevent complications, preferably starting treatment within 24 hours. Treatment depends on the cause, e.g., viral infections require antiviral medication, while noninfectious causes may include steroids, immunosuppressive agents, or biologics (infliximab, adalimumab).

The standard treatment plan for non-infectious uveitis includes:

1. *Topical Corticosteroids:* Steroid eye drops or ointments to reduce inflammation.
2. *Cycloplegic Agents:* Such as atropine or cyclopentolate to dilate the pupil and reduce pain.
3. *Systemic Immunosuppressive Medications:* In severe or recurrent cases, drugs like corticosteroids, methotrexate, or biologics may be prescribed.
4. *Pain Management:* Analgesics or nonsteroidal anti-inflammatory drugs (NSAIDs) for pain relief.
5. *Regular Ophthalmologic Monitoring:* To assess response to treatment and monitor for complications.

Treatment may vary depending on the severity and underlying cause of the uveitis and consultation with a specialist is often required.

Prognosis: The prognosis for patients with NIU varies:

- Short-term prognosis (up to 3 months) is generally favorable with appropriate treatment, as inflammation can be controlled.
- Long-term prognosis (1 year or more) may depend on the presence of complications and the underlying cause. Some patients may experience recurrent episodes, while others may achieve remission with minimal long-term sequelae.

If untreated non-infectious uveitis may lead to complications in eye such as keratopathy, synechiae, or cataract.

Differential Diagnosis: Conditions that may present similarly to NIU include:

1. *Infectious Uveitis:* Such as viral or bacterial uveitis, which require specific antimicrobial treatments.
2. *Angle-closure Glaucoma:* Elevated intraocular pressure due to blocked drainage angles.
3. *Herpetic Eye Disease:* Including herpes simplex virus (HSV) keratitis.
4. *Scleritis:* An inflammation of the sclera, which can also cause eye pain and redness.
5. *Episcleritis:* A less severe, self-limiting condition presenting with conjunctival redness.

Exclusions: Conditions or treatments that must be ruled out at the time of diagnosis include infectious causes of uveitis, systemic autoimmune diseases, and other ocular conditions that mimic the symptoms of NIU.

Ambiguity: Conditions with similar names, such as posterior uveitis or panuveitis, represent different clinical entities and involve inflammation in different parts of the uvea.

Subtypes: There are no specific subtypes of NIU; however, they can be classified based on their underlying etiology, such as idiopathic, autoimmune, or associated with systemic diseases.

References:

1. https://www.ncbi.nlm.nih.gov/books/NBK540993/
2. Jabs DA, et al. Guidelines for the Use of Immunomodulatory Drugs in Patients with Ocular Inflammatory Disorders: Recommendations of an Expert Panel. Am J Ophthalmol. 2000;130(4):492-513. https://pubmed.ncbi.nlm.nih.gov/11024423/
3. Jabs DA, et al. The Standardization of Uveitis Nomenclature (SUN) Working Group. Am J Ophthalmol. 2005;140(3):509-516. https://pubmed.ncbi.nlm.nih.gov/16196117/
4. Suhler EB, et al. A Prospective Trial of Infliximab Therapy for Refractory Uveitis: Preliminary Safety and Efficacy Outcomes. Arch Ophthalmol. 2005;123(7):903-912. https://pubmed.ncbi.nlm.nih.gov/16009830/
5. Gritz DC, et al. Incidence and Prevalence of Uveitis in Northern California: The Northern California Epidemiology of Uveitis Study. Ophthalmology. 2004;111(3):491-500. https://pubmed.ncbi.nlm.nih.gov/15019324/

Code list

Below is the concept set definition of standardized concepts used to identify NIU. When evaluated, this concept set defines a set of standard concepts and their corresponding mapped source codes. This is based on use of the OMOP Standardized Vocabularies (https://ohdsi.github.io/TheBookOfOhdsi/StandardizedVocabularies.html) and it defines one concept to represent each clinical event as “standard”. Standard concepts are often in a hierarchy, thus including descendants includes itself and any standard code that maps to it. Standard concepts and/or their children concepts can also be excluded from the definition. Standard concepts map to source codes or “non-standard concepts” (i.e., ICD9-CM, ICD10-CM) and are often what is found within the source data. Thus, the below concept set definition can be resolved within the OMOP Standardized Vocabularies and result in a list a concepts that defines the clinical idea we are interested in.

Table S1: Non-infectious uveitis and iridocyclitis concept set expression

Concept ID	Concept Name	Exclude	Descendants
4028363	Uveitis	FALSE	TRUE
4327334	Infection by Ophryoscolex	TRUE	TRUE
37311935	Infectious intermediate uveitis	TRUE	TRUE
37312551	Infectious panuveitis	TRUE	TRUE
440716	Infectious secondary iridocyclitis	TRUE	TRUE
45757573	Infectious secondary iritis	TRUE	TRUE
4132493	Infective uveitis	TRUE	TRUE
37311893	Posterior uveitis due to infectious disease	TRUE	TRUE
4109415	Secondary infected iridocyclitis	TRUE	TRUE
762971	Secondary infective uveitis	TRUE	TRUE
438961	Herpes zoster iridocyclitis	TRUE	TRUE
45770924	Herpes zoster iritis	TRUE	TRUE
440634	Herpes simplex iridocyclitis	TRUE	TRUE
439671	Uveitis due to secondary syphilis	TRUE	TRUE
4322865	Congenital syphilitic choroiditis	TRUE	TRUE
434274	Secondary syphilitic chorioretinitis	TRUE	TRUE
439731	Secondary syphilitic iridocyclitis	TRUE	TRUE
4195054	Syphilitic chorioretinitis	TRUE	TRUE
45757696	Syphilitic choroiditis	TRUE	TRUE
438971	Syphilitic disseminated retinochoroiditis	TRUE	TRUE
46273126	Syphilitic iritis	TRUE	TRUE
4196116	Tertiary syphilitic chorioretinitis	TRUE	TRUE
434926	Iridocyclitis	FALSE	TRUE
438422	Primary iridocyclitis	FALSE	TRUE
434348	Acute and subacute iridocyclitis	FALSE	TRUE
4221343	Herpetic iridocyclitis	TRUE	TRUE
45757694	Toxoplasma choroiditis	TRUE	TRUE
46273984	Cytomegalovirus chorioretinitis	TRUE	TRUE
761322	Iritis of left eye caused by herpes simplex virus	TRUE	TRUE
37209448	Iritis of left eye caused by herpes zoster virus	TRUE	TRUE
761323	Iritis of right eye caused by herpes simplex virus	TRUE	TRUE
37209447	Iritis of right eye caused by herpes zoster virus	TRUE	TRUE
4072340	Herpes simplex iritis	TRUE	TRUE
4234762	Parasitic chorioretinitis	TRUE	TRUE
4230365	Parasitic choroiditis	TRUE	TRUE
4265441	Herpes simplex keratouveitis	TRUE	TRUE
761312	Chorioretinitis of bilateral eyes caused by Toxoplasma gondii	TRUE	TRUE
761311	Chorioretinitis of left eye caused by Toxoplasma gondii	TRUE	TRUE
761313	Chorioretinitis of right eye caused by Toxoplasma gondii	TRUE	TRUE
4230371	Fungal chorioretinitis	TRUE	TRUE
4230369	Fungal choroiditis	TRUE	TRUE
36687174	Uveitis of bilateral eyes caused by Toxoplasma gondii	TRUE	TRUE
36687176	Uveitis of left eye caused by Toxoplasma gondii	TRUE	TRUE
36687175	Uveitis of right eye caused by Toxoplasma gondii	TRUE	TRUE
438751	Lens-induced iridocyclitis	TRUE	FALSE
438739	Sympathetic uveitis	TRUE	FALSE
434932	Fuchs' heterochromic cyclitis	TRUE	FALSE
432908	Glaucomatocyclitic crisis	TRUE	FALSE
46269950	Iridocyclitis due to tuberculosis	TRUE	FALSE
438744	Acute posterior multifocal placoid pigment epitheliopathy	TRUE	FALSE
440715	Parasitic endophthalmitis	TRUE	FALSE

Candidate outcome definitions

Broad

Cohort Entry Events

People enter the cohort when observing any of the following:

- condition occurrence of 'Non-infectious uveitis or iridocyclitis' for the first time in the person's history.

Limit cohort entry events to the earliest event per person.

Cohort Exit

The person exits the cohort at the end of continuous observation.

Narrow

Cohort Entry Events

People enter the cohort when observing any of the following:

- condition occurrence of 'Non-infectious uveitis or iridocyclitis' for the first time in the person's history; having at least 1 condition occurrence of 'Non-infectious uveitis or iridocyclitis', starting between 31 days after and 365 days after 'Non-infectious uveitis or iridocyclitis' start date.

Limit cohort entry events to the earliest event per person.

Cohort Exit

The person exits the cohort at the end of continuous observation.

Primary

Cohort Entry Events

People enter the cohort when observing any of the following:

- condition occurrence of 'Non-infectious uveitis or iridocyclitis' for the first time in the person's history; having at least 1 condition occurrence of 'Non-infectious uveitis or iridocyclitis', starting between 31 days after and 365 days after 'Non-infectious uveitis or iridocyclitis' start date.
- condition occurrence of 'Non-infectious uveitis or iridocyclitis' for the first time in the person's history; having at least 1 visit occurrence of any visit, starting anytime on or before 'Non-infectious uveitis or iridocyclitis' start date and ending between 0 days after and all days after 'Non-infectious uveitis or iridocyclitis' start date; a provider specialty that is: "ophthalmology", "retina ophthalmology", "glaucoma ophthalmology", "pediatric ophthalmology and strabismus", "cornea and external ophthalmology", "paediatric ophthalmology", "neuro-ophthalmology", "uveitis and ocular inflammatory disease ophthalmology" or "orthoptics".

Limit cohort entry events to the earliest event per person.

Cohort Exit

The person exits the cohort at the end of continuous observation.

Figure 1 presents a visual representation of the Primary outcome definition: [first occurrence of a NIU code with a second NIU code occurrence between 31 days and 365 days relative to first occurrence] OR [first occurrence of a NIU code during an ophthalmology visit]

Figure S1. Schematic of primary outcome cohort definition for non-infectious uveitis outcome definition

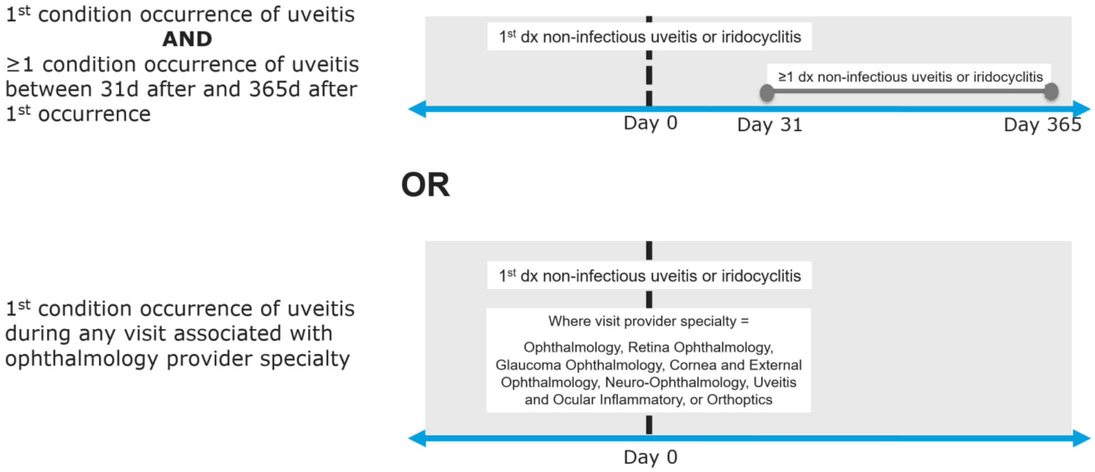

Phenotype development and evaluation results

Characterization results from five databases on the three candidate outcome cohorts are available at https://results.ohdsi.org/app/14_UveitisSafetyOutcomes. We compared the clinical characteristics of patients returned by the Primary definition to the Broad definition and the Narrow definition. Below are some observations we made while reviewing these characterization results.

Cohort counts

Figure S2. Broad, Narrow, and Primary Non-infectious uveitis (NIU) outcome cohort counts across five databases

			iqvia_amb_emr	iqvia_pharmetrics_plus	optum_ehr	optum_extended_dod	truven_ccae
	Cohort Id	Cohort Name	Persons	Persons	Persons	Persons	Persons
○	8466	Non-infectious uveitis (primary)	25,626	176,396	71,977	260,779	292,132
○	8467	Non-infectious uveitis (narrow)	17,430	157,411	38,578	120,796	135,559
○	8468	Non-infectious uveitis (broad)	51,480	480,305	145,863	385,359	470,604

Figure 2 reports the cohort counts. The Broad definition returned the most patients across all data sources and the narrow definition returned the fewest. The Primary definition included all patients from the narrow definition but only those from the Broad definition whose NIU code was observed in an ophthalmology visit were also include. Hence, Primary definition patient counts were greater than that of the Narrow definition but less than that of the Broad definition. The single code allowance during an ophthalmology visit returned patients that were excluded from the more restrictive Narrow definition. For example, in CCAE, the Primary definition returned 292,132 patients, whereas the Narrow returned 135,559 and the Broad 470,604.

Cohort overlap

Patient overlap between the Primary and Narrow definitions across database ranged from 46% (CCAE, Clinformatics®) to 89% (Pharmetrics). Conversely, the additional patients in the Primary definition ranged from 11% (Pharmetrics) to 54% (CCAE, Clinformatics®).

Background incidence

Figure 3 reports the incidence of NIU among patients with ≥365 days of observation time stratified by age, sex, and calendar year. Background incidence was generally low and ranged from 0.09 to 0.92/1000 person-years (PY), which corresponds to 'very rare' to 'rare' per the Council for International Organizations of Medical Sciences (CIOMS) categorization of frequency of adverse drug reactions [1]). Background incidence was generally greater in women and increases with age. Incidence was mostly stable over time and there was little qualitative indication of an incidence increase or decrease in 2015 when US data sources switch from ICD9-CM to ICD10-CM coding standards. Lastly, outlying high incidence rates in earlier or later years reflect statistical instability related to incomplete data capture during partial years (e.g., see rate among males aged 20-29 years in 2021).

Figure S3. Background incidence rate of NIU (Primary definition) stratified by sex, year, age, and database

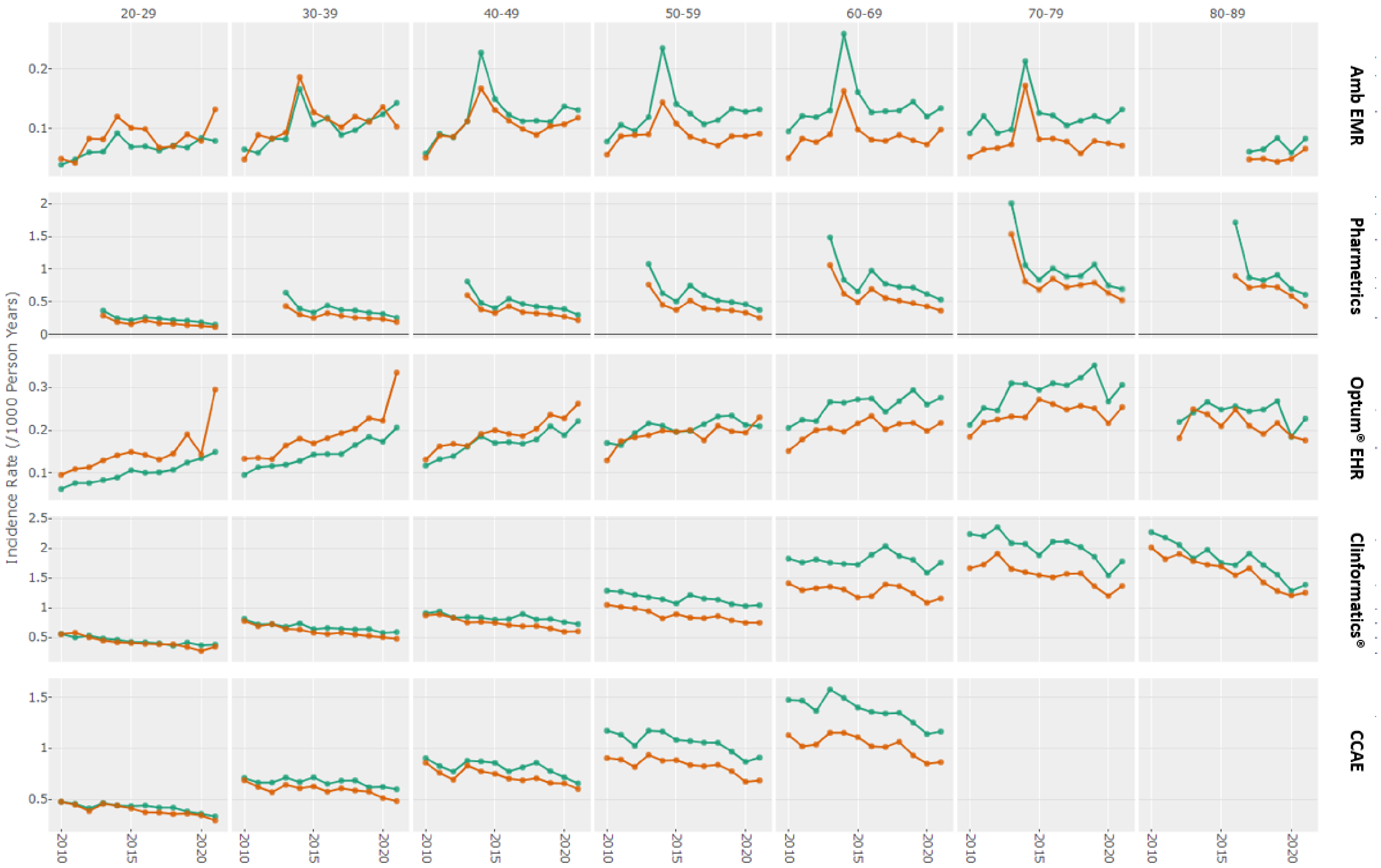

Key – green data points: female, orange data points: male

Index event breakdown

The most common standardized concepts at index in all databases, except Amb EMR, were Iridocyclitis, Acute and subacute iridocyclitis, and Primary iridocyclitis. Patients experienced these codes 25.5%-52.3%, 17.8%-24.5%, and 7.0%-22.1% of the time at index. Amb EMR's top codes were Iritis (20.2%), Uveitis (14.3%), and Iridocyclitis (11.2%).

Characterization

When the primary definition is characterized for events before index (-30 to -1 days) we see evidence of the sign and symptoms described in the clinical description. Concepts like "conjunctivitis", "visual disturbance", and "pain in eye" are suggestive of clinical events related to NIU and are seen prior to index. On the day of index, we see clinical concepts related to our definition of NIU and we did not see evidence of our differential diagnoses (e.g., infectious uveitis) as this may suggest we are pulling in conditions that present similarly to NIU. Also, day of index we see patients picking up scripts for steroids suggesting the physician is treating the NIU. However, we do also see exposures to anti-infectives, suggesting that physicians are covering both possible outcomes of infectious uveitis and NIU. Also, day of index we see evidence of eye related procedures, like "Fundus photography with interpretation and report". What we are seeing within the characterization is in line with the clinical description.

Validation

Table S2: Confusion matrix contingency cell counts and misclassification errors for the broad, narrow, and primary outcome definitions across databases

Database	NIU outcome definition	TP	TN	FP	FN	Sensitivity	Specificity	PPV	NPV
Amb EMR	Broad	945	1412318	516	17	0.98231	0.999635	0.646133	0.999988
Amb EMR	Narrow	360	1412617	217	602	0.37461	0.999846	0.623917	0.999574
Amb EMR	Primary	496	1412594	241	465	0.516129	0.999829	0.672999	0.99967
Pharmetrics	Broad	5800	1965431	3778	2827	0.672192	0.998081	0.60545	0.998563
Pharmetrics	Narrow	2091	1968056	1153	6536	0.242379	0.999415	0.644575	0.99669
Pharmetrics	Primary	2249	1967965	1244	6378	0.260577	0.999368	0.643573	0.996769
Optum® EHR	Broad	2733	1946194	826	1907	0.589009	0.999576	0.767912	0.999021
Optum® EHR	Narrow	839	1946842	177	3802	0.180603	0.999909	0.824803	0.998051
Optum® EHR	Primary	1369	1946703	317	3271	0.295043	0.999838	0.811981	0.998323
Clinformatics®	Broad	8646	1657041	2639	4086	0.679051	0.99841	0.766061	0.99754
Clinformatics®	Narrow	3497	1658857	824	9234	0.274684	0.999504	0.809303	0.994464
Clinformatics®	Primary	6007	1658007	1673	6725	0.471762	0.998992	0.782031	0.99596
CCAE	Broad	6874	1911524	1870	3454	0.665537	0.999023	0.786025	0.998196
CCAE	Narrow	2423	1912944	450	7905	0.234531	0.999765	0.843021	0.995885
CCAE	Primary	4044	1912446	949	6283	0.391595	0.999504	0.809934	0.996725

Key – Amb EMR: IQVIA Ambulatory Electronic Medical Records, Pharmetrics: IQVIA Adjudicated Health Plan Claims Data, Optum® EHR: Optum® De-Identified Electronic Health Record, Clinformatics®: Optum® De-Identified Clinformatics® Data Mart Database, CCAE: Merative™ MarketScan® Commercial Database, TP: true positives, TN: true negatives, FP: false positives, FN: false negatives, Sensitivity = TP/(TP+FN), Specificity = TN/(TN+FP), PPV = positive predictive value = TP/(TP+FP); NPV = negative predictive value = TN/(TN+FN), Broad: first occurrence of a NIU code, Narrow: first occurrence of a NIU code with a second NIU code occurrence between 31 days and 365 days relative to first occurrence, Primary: [first occurrence of a NIU code with a second NIU code occurrence between 31 days and 365 days relative to first occurrence] OR [first occurrence of a NIU code during an ophthalmology visit]

Three NIU algorithms were analyzed using probabilistic reference standard validation across five databases. Overall, sensitivity ranged from 18-98% and positive predictive value (PPV) from 61-84% (Table 2). Both specificity and negative predictive value (NPV) were above 99% regardless of database or NIU algorithm. The highest average sensitivities across the databases were for the Broad definition at 72% (range 59-98%) and the lowest for the Narrow definition at 26% (range 18-37%). The highest average PPVs across databases was for the Narrow definition at 75% (range 62-84%) and the lowest for Broad at 71% (range 61%-79%).

In comparing our selected Primary definition versus our Broad and Narrow definitions, we saw that broad typically had the highest sensitivity, Narrow the lowest, and Primary tended to recover the Narrow sensitivity. In terms of PPV, it was the lowest for Broad and typically highest for Narrow. However, we chose Primary as it recovers some of the average sensitivity that is lost in Narrow (39% vs 26%) and its average PPV is not much lower (74% Primary vs 75% Narrow).

Appendix 4: Covariates for propensity score model inclusion and PS matching balance assessment

- Demographics (age in 5-year bands, sex, race, ethnicity, index year, index month)
- All conditions occurrence records aggregated to SNOMED clinical finding level during the following lookback windows:
    - in 365 days prior to and including index date
    - in 30 days prior to and including index date
- All drug exposure records aggregated to RxNorm ingredient level and Anatomical Therapeutic Chemical code (ATC) classes during the following lookback windows:
    - in 365 days prior to and including index date
    - in 30 days prior to and including index date
    - persistent exposure that overlaps index date
- All procedure occurrence records during the following lookback windows:
    - in 365 days prior to and including index date
    - in 30 days prior to and including index date
- Measurements (including laboratories) within, above, and below normal range during the following lookback window:
    - in 365 days prior to and including index date
- Device exposure records during the following lookback windows:
    - in 365 days prior to and including index date
    - in 30 days prior to and including index date
- Comorbidity or risk scores described below are calculated using all available time prior to index date:
    - Charlson Comorbidity Index[2]
    - DCSI[3]
    - CHADS2[4]
    - CHADS2VASc[5]

Appendix 5: Time between subsequent exposures to study drugs

Table S3. Distribution in days between exposure date to the next exposure date for study drug exposure records in 3 databases used in the study

Database	Exposure name	Records	Mean	Std. Dev.	Min	p10	p25	p50	p75	p90	Max
CCAE	certolizumab pegol	256231	39.6	50.3	1	22	26	30	37	70	3525
	golimumab	217030	46.9	59.0	1	25	28	34	57	81	3654
	guselkumab	36948	62.8	44.5	1	37	49	56	65	85	1025
	infliximab	1716962	54.4	72.3	1	28	43	52	56	66	6733
	infliximab	1716962	54.4	72.3	1	28	43	51	57	65	6733
	Remicade	107258	56.6	65.1	1	29	41	52	62	79	4222
	secukinumab	199690	39.0	40.7	1	22	25	29	38	69	1714
	ustekinumab	259800	81.0	74.9	1	28	51	77	93	117	3232
	vedolizumab	158616	48.5	37.4	1	25	28	55	57	64	1839
Optum® EHR	certolizumab pegol	132724	64.2	135.1	1	5	14	28	63	145	3419
	golimumab	103972	67.9	133.6	1	4	15	36	65	143	3785
	guselkumab	18077	71.7	104.2	1	6	15	44	82	175	3813
	infliximab	1047419	55.3	137.4	1	3	14	38	57	74	4855
	infliximab	1047419	55.3	137.4	1	4	14	38	56	73	4855
	Remicade	831118	62.7	153.1	1	6	15	39	56	93	4867
	secukinumab	114350	58.5	94.8	1	4	14	28	66	138	2317
	ustekinumab	167901	79.1	143.1	1	4	13	40	91	181	3886
	vedolizumab	162656	44.5	79.8	1	4	12	29	56	64	3187
Clinformatics®	certolizumab pegol	238101	37.1	61.6	1	22	27	30	35	52	4469
	golimumab	175961	44.1	73.9	1	24	29	33	54	62	4051
	guselkumab	28617	62.8	47.5	1	37	46	56	66	89	1037
	infliximab	1029840	58.4	124.8	1	29	43	54	56	67	6732
	infliximab	1029840	58.4	124.8	1	29	43	54	56	67	6732
	Remicade	409159	60.6	114.6	1	28	43	54	57	71	5236
	secukinumab	144707	35.2	38.2	1	22	24	28	36	49	1859
	ustekinumab	162133	83.3	101.5	1	30	50	75	92	117	3813
	vedolizumab	113102	49.5	47.0	1	26	28	56	56	62	1993

CCAE: Merative™ MarketScan® Commercial Database, Optum® EHR: Optum® De-Identified Electronic Health Record, Clinformatics®: Optum® De-Identified Clinformatics® Data Mart Database

Appendix 6: Negative control outcomes

Table S4. The following conditions are assumed to not to be causally associated with the target or comparator exposures on the basis of spontaneous reports, published literature, and product labels.

Concept ID	Outcome name
201826	Type 2 diabetes mellitus
321689	Apnea
313459	Sleep apnea
4209423	Nicotine dependence
442588	Obstructive sleep apnea syndrome
138384	Acquired hypothyroidism
437833	Hypokalemia
133729	Hyperparathyroidism
373769	Nuclear cataract
201254	Type 1 diabetes mellitus
374375	Impacted cerumen
437246	Vitamin B deficiency
4308509	Impaired fasting glycemia
433163	Deficiency of macronutrients
434610	Hyperkalemia
201916	Ureteric stone
4156515	Malnutrition (calorie)
438134	Hypersomnia
443800	Amenorrhea
200528	Ascites
73754	Restless legs
4142875	Solitary nodule of lung
193326	Urge incontinence of urine
40304526	Nocturia
4302555	Menorrhagia
377575	Tinnitus
381877	Dysfunction of eustachian tube
433527	Endometriosis (clinical)
199075	Neurogenic bladder
440129	Hypertrophy of nasal turbinates
4098604	Hypomagnesemia
377888	Conductive hearing loss
440276	Infection AND/OR inflammatory reaction due to internal prosthetic device, implant AND/OR graft
197607	Excessive and frequent menstruation
434004	Hypervolemia
253796	Pneumothorax
436940	Metabolic syndrome X
73560	Calcaneal spur
434002	Mineral deficiency
441829	Hyperosmolality and or hypernatremia
195873	Leukorrhea
257683	Posterior rhinorrhea
4152350	Burping
40326053	Dysphonia
443524	Mixed urinary incontinence
4196636	Dysarthria

Concept ID	Outcome name
197675	Incontinence of feces
4146209	Iron deficiency
437969	Talipes planus
374367	Bilateral hearing loss
195590	Urethral stricture
79833	Meniere's disease
195862	Urethritis
317376	Tachypnea
195012	Intermenstrual bleeding - irregular
434821	Systemic inflammatory response syndrome
439081	Premenstrual tension syndrome
134765	Cachexia
4143972	Raynaud's phenomenon
4209859	Empyema
74052	Labyrinthitis
439794	Central sleep apnea syndrome
4225324	Resistance to activated protein C due to Factor V Leiden
4299544	Acanthosis nigricans
4305841	Periodic limb movement disorder
136516	Congenital pes planus
374053	Sudden hearing loss
441553	Myoclonus
4241033	Acute abdomen
4181744	Discharge from nipple
198802	Occlusion of ureter
197239	Hemangioma of intra-abdominal structure
75311	Fibrosclerosis of breast
316814	Hyperventilation
436093	Disseminated intravascular coagulation
381033	Aphakia
194475	Hiccoughs
194408	Hydroureter
4192647	Hypovolemic shock
4152370	Iodine-deficiency-related endemic goiter
261116	Acquired deformity of nose
432508	Mechanical complication of nervous system device, implant AND/OR graft
198715	Premature menopause
437241	Qualitative platelet disorder
133327	Viremia
434910	Monocytosis

Appendix 7: Evidence validity diagnostics for secondary analyses

Diagnostic results for secondary analyses, rows shaded blue passed 4 validity diagnostics

Study pop.	Analysis	Database	Target	Comparator	T events	C events	0 event Pass	Max ASD	ASD Pass	Equipoise	Equipoise Pass	EASE	EASE Pass	Total Passed
AS	S1	Amb EMR	Remicade®	AS comp.	<5	<5	1	0.302	0	0.535	1	0.128	1	3
AS	S1	Pharmetrics	Remicade®	AS comp.	8	18	1	0.157	0	0.405	1	0.095	1	3
AS	S1	Optum® EHR	Remicade®	AS comp.	12	18	1	0.265	0	0.635	1	0.024	1	3
AS	S1	Clinformatics®	Remicade®	AS comp.	6	7	1	0.272	0	0.417	1	0.167	1	3
AS	S1	CCAE	Remicade®	AS comp.	9	16	1	0.239	0	0.434	1	0.134	1	3
AS	S2	Amb EMR	Remicade®	AS comp.	0	0	0	0.286	0	0.531	1	0.693	0	1
AS	S2	Pharmetrics	Remicade®	AS comp.	6	8	1	0.19	0	0.405	1	0.050	1	3
AS	S2	Optum® EHR	Remicade®	AS comp.	<5	<5	1	0.218	0	0.638	1	0.222	1	3
AS	S2	Clinformatics®	Remicade®	AS comp.	<5	<5	1	0.313	0	0.417	1	0.139	1	3
AS	S2	CCAE	Remicade®	AS comp.	<5	<5	1	0.292	0	0.434	1	0.451	0	2
AS	S3	Amb EMR	Remicade®	AS comp.	<5	0	0	0.277	0	0.535	1	0.209	1	2
AS	S3	Pharmetrics	Remicade®	AS comp.	8	15	1	0.19	0	0.405	1	0.107	1	3
AS	S3	Optum® EHR	Remicade®	AS comp.	12	10	1	0.245	0	0.635	1	0.052	1	3
AS	S3	Clinformatics®	Remicade®	AS comp.	6	5	1	0.313	0	0.417	1	0.120	1	3
AS	S3	CCAE	Remicade®	AS comp.	9	7	1	0.292	0	0.434	1	0.082	1	3
IBD	S1	Amb EMR	Remicade®	IBD comp.	<5	<5	1	0.082	1	0.422	1	0.008	1	4
IBD	S1	Pharmetrics	Remicade®	IBD comp.	27	68	1	0.047	1	0.431	1	0.099	1	4
IBD	S1	Optum® EHR	Remicade®	IBD comp.	18	26	1	0.058	1	0.481	1	0.147	1	4
IBD	S1	Clinformatics®	Remicade®	IBD comp.	18	40	1	0.105	0	0.412	1	0.085	1	3
IBD	S1	CCAE	Remicade®	IBD comp.	23	41	1	0.068	1	0.386	1	0.153	1	4
IBD	S2	Amb EMR	Remicade®	IBD comp.	<5	<5	1	0.089	1	0.422	1	0.241	1	4
IBD	S2	Pharmetrics	Remicade®	IBD comp.	12	30	1	0.047	1	0.431	1	0.105	1	4
IBD	S2	Optum® EHR	Remicade®	IBD comp.	<5	5	1	0.055	1	0.480	1	0.156	1	4
IBD	S2	Clinformatics®	Remicade®	IBD comp.	6	11	1	0.107	0	0.412	1	0.069	1	3
IBD	S2	CCAE	Remicade®	IBD comp.	10	11	1	0.072	1	0.387	1	0.122	1	4
IBD	S3	Amb EMR	Remicade®	IBD comp.	<5	<5	1	0.086	1	0.422	1	0.004	1	4
IBD	S3	Pharmetrics	Remicade®	IBD comp.	27	50	1	0.047	1	0.431	1	0.126	1	4
IBD	S3	Optum® EHR	Remicade®	IBD comp.	18	23	1	0.058	1	0.481	1	0.147	1	4
IBD	S3	Clinformatics®	Remicade®	IBD comp.	18	29	1	0.107	0	0.412	1	0.118	1	3
IBD	S3	CCAE	Remicade®	IBD comp.	23	30	1	0.07	1	0.386	1	0.165	1	4
PsO/PsA	S1	Amb EMR	Remicade®	PsO/PsA comp.	0	5	0	0.148	0	0.255	0	0.129	1	1
PsO/PsA	S1	Pharmetrics	Remicade®	PsO/PsA comp.	8	19	1	0.132	0	0.155	0	0.099	1	2
PsO/PsA	S1	Optum® EHR	Remicade®	PsO/PsA comp.	13	17	1	0.101	0	0.305	0	0.007	1	2
PsO/PsA	S1	Clinformatics®	Remicade®	PsO/PsA comp.	<5	16	1	0.199	0	0.171	0	0.009	1	2
PsO/PsA	S1	CCAE	Remicade®	PsO/PsA comp.	6	16	1	0.167	0	0.147	0	0.017	1	2
PsO/PsA	S2	Amb EMR	Remicade®	PsO/PsA comp.	0	0	0	0.166	0	0.254	0	0.830	0	0
PsO/PsA	S2	Pharmetrics	Remicade®	PsO/PsA comp.	<5	<5	1	0.144	0	0.155	0	0.136	1	2
PsO/PsA	S2	Optum® EHR	Remicade®	PsO/PsA comp.	6	<5	1	0.137	0	0.306	0	0.203	1	2
PsO/PsA	S2	Clinformatics®	Remicade®	PsO/PsA comp.	0	<5	0	0.211	0	0.171	0	0.020	1	1
PsO/PsA	S2	CCAE	Remicade®	PsO/PsA comp.	<5	<5	1	0.195	0	0.147	0	0.068	1	2
PsO/PsA	S3	Amb EMR	Remicade®	PsO/PsA comp.	0	<5	0	0.181	0	0.255	0	0.025	1	1
PsO/PsA	S3	Pharmetrics	Remicade®	PsO/PsA comp.	8	10	1	0.144	0	0.155	0	0.031	1	2

Study pop.	Analysis	Database	Target	Comparator	T events	C events	0 event Pass	Max ASD	ASD Pass	Equipoise	Equipoise Pass	EASE	EASE Pass	Total Passed
PsO/PsA	S3	Optum® EHR	Remicade®	PsO/PsA comp.	13	<5	1	0.112	0	0.305	0	0.000	1	2
PsO/PsA	S3	Clinformatics®	Remicade®	PsO/PsA comp.	<5	6	1	0.211	0	0.171	0	0.061	1	2
PsO/PsA	S3	CCAE	Remicade®	PsO/PsA comp.	6	6	1	0.195	0	0.147	0	0.044	1	2
RA	S1	Amb EMR	Remicade®(m)	RA comp.	6	<5	1	0.129	0	0.448	1	0.110	1	3
RA	S1	Pharmetrics	Remicade®(m)	RA comp.	<5	9	1	0.179	0	0.352	1	0.085	1	3
RA	S1	Optum® EHR	Remicade®(m)	RA comp.	10	21	1	0.086	1	0.557	1	0.063	1	4
RA	S1	Clinformatics®	Remicade®(m)	RA comp.	8	14	1	0.252	0	0.363	1	0.018	1	3
RA	S1	CCAE	Remicade®(m)	RA comp.	<5	14	1	0.151	0	0.508	1	0.026	1	3
RA	S2	Amb EMR	Remicade®(m)	RA comp.	<5	0	0	0.17	0	0.445	1	0.257	0	1
RA	S2	Pharmetrics	Remicade®(m)	RA comp.	<5	0	0	0.189	0	0.352	1	0.149	1	2
RA	S2	Optum® EHR	Remicade®(m)	RA comp.	5	<5	1	0.096	1	0.558	1	0.049	1	4
RA	S2	Clinformatics®	Remicade®(m)	RA comp.	<5	<5	1	0.268	0	0.363	1	0.225	1	3
RA	S2	CCAE	Remicade®(m)	RA comp.	4	5	1	0.18	0	0.508	1	0.078	1	3
RA	S3	Amb EMR	Remicade®(m)	RA comp.	6	<5	1	0.145	0	0.448	1	0.118	1	3
RA	S3	Pharmetrics	Remicade®(m)	RA comp.	<5	<5	1	0.189	0	0.352	1	0.054	1	3
RA	S3	Optum® EHR	Remicade®(m)	RA comp.	10	8	1	0.093	1	0.557	1	0.083	1	4
RA	S3	Clinformatics®	Remicade®(m)	RA comp.	8	7	1	0.268	0	0.363	1	0.120	1	3
RA	S3	CCAE	Remicade®(m)	RA comp.	<5	8	1	0.18	0	0.508	1	0.040	1	3

Key – <5: a censored value between 1 and 4, Amb EMR: IQVIA Ambulatory Electronic Medical Records, AS comparator: [certolizumab pegol, golimumab, ixekizumab, or secukinumab], AS: ankylosing spondylitis, ASD: absolute standardized difference, CCAE: IBM® MarketScan® Commercial Claims and Encounters Database, Clinformatics®: Optum® De-Identified Clinformatics® Data Mart Database, EASE: expected absolute systematic error, IBD comparator: [golimumab, certolizumab pegol, ustekinumab, or vedolizumab], IBD: inflammatory bowel diseases (Crohn's disease or ulcerative colitis), Optum® EHR: Optum® De-Identified Electronic Health Record, Pharmetrics: IQVIA Adjudicated Health Plan Claims Data, PsO/PsA comparator: [golimumab, certolizumab pegol, guselkumab, risankizumab, tildrakizumab, brodalumab, ixekizumab, secukinumab, or ustekinumab], PsO/PsA: psoriatic conditions plaque psoriasis or psoriatic arthritis, RA comparator: certolizumab pegol or tocilizumab, RA: rheumatoid arthritis; Remicade®(m): Remicade® exposure with concurrent methotrexate, Study pop.: study population, S: secondary analysis, S1: [1:1 PS matched, ITT], S2: [1:1 PS matched on-treatment]

References

1. *Guidelines for Preparing Core Clinical-Safety Information on Drugs Second Edition – Report of CIOMS Working Groups III and V*. 1999.
2. Charlson, M.E., et al., *A new method of classifying prognostic comorbidity in longitudinal studies: development and validation.* J Chronic Dis, 1987. **40**(5): p. 373-83.
3. Young, B.A., et al., *Diabetes complications severity index and risk of mortality, hospitalization, and healthcare utilization.* Am J Manag Care, 2008. **14**(1): p. 15-23.
4. Gage, B.F., et al., *Validation of clinical classification schemes for predicting stroke: results from the National Registry of Atrial Fibrillation.* Jama, 2001. **285**(22): p. 2864-70.
5. Lip, G.Y., et al., *Refining clinical risk stratification for predicting stroke and thromboembolism in atrial fibrillation using a novel risk factor-based approach: the euro heart survey on atrial fibrillation.* Chest, 2010. **137**(2): p. 263-72.
